# Obesity defined molecular endotypes in the synovium of patients with osteoarthritis provides a rationale for therapeutic targeting of fibroblast subsets

**DOI:** 10.1002/ctm2.1232

**Published:** 2023-04-03

**Authors:** Susanne N. Wijesinghe, Amel Badoume, Dominika E. Nanus, Archana Sharma‐Oates, Hussein Farah, Michelangelo Certo, Fawzeyah Alnajjar, Edward T. Davis, Claudio Mauro, Mark A. Lindsay, Simon W. Jones

**Affiliations:** ^1^ Institute of Inflammation and Ageing MRC‐ARUK Centre for Musculoskeletal Ageing Research University of Birmingham Birmingham UK; ^2^ Department of Pharmacy and Pharmacology University of Bath, Claverton Down Bath UK; ^3^ School of Biosciences University of Birmingham Birmingham UK; ^4^ The Royal Orthopaedic Hospital Birmingham UK

**Keywords:** molecular endotypes, obesity, osteoarthritis, synovial fibroblasts

## Abstract

**Background:**

Osteoarthritis (OA), a multifaceted condition, poses a significant challenge for the successful clinical development of therapeutics due to heterogeneity. However, classifying molecular endotypes of OA pathogenesis could provide invaluable phenotype‐directed routes for stratifying subgroups of patients for targeted therapeutics, leading to greater chances of success in trials. This study establishes endotypes in OA soft joint tissue driven by obesity in both load‐bearing and non‐load bearing joints.

**Methods:**

Hand, hip, knee and foot joint synovial tissue was obtained from OA patients (*n* = 32) classified as obese (BMI > 30) or normal weight (BMI 18.5–24.9). Isolated fibroblasts (OA SF) were assayed by Olink proteomic panel, seahorse metabolic flux assay, Illumina's NextSeq 500 bulk and Chromium 10X single cell RNA‐sequencing, validated by Luminex and immunofluorescence.

**Results:**

Targeted proteomic, metabolic and transcriptomic analysis found the inflammatory landscape of OA SFs are independently impacted by obesity, joint loading and anatomical site with significant heterogeneity between obese and normal weight patients, confirmed by bulk RNAseq. Further investigation by single cell RNAseq identified four functional molecular endotypes including obesity specific subsets defined by an inflammatory endotype related to immune cell regulation, fibroblast activation and inflammatory signaling, with up‐regulated CXCL12, CFD and CHI3L1 expression. Luminex confirmed elevated chitase3‐like‐1(229.5 vs. 49.5 ng/ml, *p* < .05) and inhibin (20.6 vs. 63.8 pg/ml, *p* < .05) in obese and normal weight OA SFs, respectively. Lastly, we find SF subsets in obese patients spatially localise in sublining and lining layers of OA synovium and can be distinguished by differential expression of the transcriptional regulators MYC and FOS.

**Conclusion:**

These findings demonstrate the significance of obesity in changing the inflammatory landscape of synovial fibroblasts in both load bearing and non‐load bearing joints. Describing multiple heterogeneous OA SF populations characterised by specific molecular endotypes, which drive heterogeneity in OA disease pathogenesis. These molecular endotypes may provide a route for the stratification of patients in clinical trials, providing a rational for the therapeutic targeting of specific SF subsets in specific patient populations with arthritic conditions.

## Graphical abstract

The inflammatory landscape of OA synovial fibroblasts (SFs), from hand, hip, knee and foot joint synovial tissue, are independently impacted by obesity, joint loading, and anatomical site. Resulting in significant heterogeneity between obese and normal weight patients, due to the presence and predominance of specific SF subsets, which can be defined by four functional molecular endotypes.

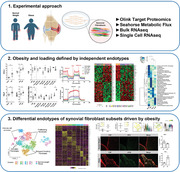

## 1 INTRODUCTION

Osteoarthritis (OA) is a major cause of disability and globally the most common musculoskeletal health issue with >30% of those over 45 years of age having sought treatment.^1^, ^2^, ^3^ Despite this, there are currently no approved disease‐modifying drugs and only generic analgesic treatments, which are of limited efficacy and associated with toxicity over long term use. Consequently, the disease in many patients will progress, both in terms of structural damage and pain, before patients require joint replacement surgery, a procedure with a high percentage of poor post‐operative outcome.^4^ In recent attempts to develop new treatments for patients it is now emerging that the multifaceted clinical pathology of OA is underpinned by particular molecular endotypes defined by distinct molecular mechanisms and signaling pathways, which may overlap. In OA these include ‘low repair’, ‘bone cartilage’, ‘metabolic’ and ‘inflammatory’ endotypes.^5^ Importantly, the inflammatory endotype, as reported by Angelini 2022, contains the highest proportion of pain‐related progressors, correlating with patients who progress both in pain and in structural joint damage.^6^ Therefore, better understanding of this inflammatory endotype represents an opportunity for developing targeted therapeutics that reduce both disease progression and joint pain.

In attempting to understand the drivers of these endotypes, it is notable that obesity is a major risk factor for the development of OA. A systematic review and meta‐analysis of risk factors for knee OA found obesity to be one of the main risk factors, alongside previous knee trauma,^7^ whilst two meta‐analyses by Jiang et al., 2011 and 2012 found a 5‐unit increase in BMI was associated with a 35% increased risk of knee OA and an 11% increased risk of developing hip OA.^8^, ^9^ Importantly, obesity is also associated with greater functional impairment in patients with knee OA,^10^ increased musculoskeletal pain^11^ and reduced responsiveness to therapeutic agents in patients with rheumatoid arthritis.^12^ Excessive mechanical loading of the joint is often cited as the cause of the association between obesity and OA. However, studies also find that obesity increases the risk of developing OA in the hands,^13^ a non‐load bearing joint, illustrating that the association is not solely due to pathological loading on the articular cartilage but may also be due to the chronic inflammatory metabolic effects of obesity. Indeed, we have reported that in patients with hip OA, obesity exacerbates one of the key hallmarks of OA, namely synovial inflammation (synovitis). Synovitis, characterized by hyperplasia of the resident synovial fibroblasts (SFs) and the infiltration of innate immune cells, increases the secretion of pro‐inflammatory cytokines into the synovial fluid driving matrix metalloprotease (MMP) and aggrecanase‐induced cartilage damage and promoting joint pain by sensitizing peripheral nociceptors.^1^, ^2^, ^14^, ^15^, ^16^, ^17^ In our previous studies, we found that the synovial joint fluid in obese OA patients contains significantly greater levels of pro‐inflammatory cytokines, including IL6, CXCL8 and TNFα, compared to patients of normal weight.^18^ Furthermore, we found that obesity imparts an inflammatory SF phenotype, with SFs from obese patients being more proliferative and exhibiting an inflammatory transcriptome, compared to fibroblasts from normal‐weight patients,^16^ including the expression of several long non‐coding RNAs that are found to be associated with obesity‐related inflammatory musculoskeletal disorders.^19^ This is important as recent studies suggest the inflammatory status of the synovial joint lining tissue is reflected in the imprinted phenotype of the SF, which maintains a stable phenotype and epigenetic modifications such as DNA methylation in culture.^20^, ^21^ The heterogeneity of inflammatory synovial tissue being reflected in SFs as a stable trait has led to much focus on the role of SFs in mediating joint inflammation. Recent findings suggest there are functionally distinct populations of SFs,^22^ which differentially contribute to the severity of arthritis,^23^ and to the severity of joint pain.^2^, ^18^, ^24^ Although whether such populations are impacted by obesity is yet to be determined.

Understanding the nature of inflammatory SF phenotype in the context of obesity and joint loading may therefore help to better understand the metabolic and inflammatory endotypes in OA, which ultimately will facilitate stratification of patients for clinical trials and help to identify targets for the development of new therapeutics. As such, the aim of this study was to determine the confounding impact of obesity and loading on OA pathogenesis, comparing load‐bearing and non‐load bearing joints, to identify differences in SF secretomes, transcriptomes and cellular subsets that exhibit pathologically inflammatory functions driven by obesity.

## 2 PATIENTS AND METHODS

### 2.1 Study recruitment

Ethical approval was granted by the UK National Research Ethics Committee (NRES 16/SS/0172) to collect OA joint tissue from consenting patients following elective total joint replacement. Patients were recruited to the study at the Royal Orthopaedic Hospital, Birmingham (United Kingdom) and Russell's Hall Hospital, Dudley (United Kingdom).

### 2.2 Primary SF isolation and characterization

Synovial joint tissue (*n* = 32) was collected peri‐operatively and used to isolate and characterise primary SF as previously described.^16^, ^24^, ^25^ Each joint sample collected was from an independent patient undergoing arthroscopy at a single anatomical joint, see Supplementary Materials (SM. 1) for the summary of patient characteristics. Fibroblasts were conditioned in low serum (1% fetal bovine serum) media (*n* = 24), which was collected after 24 h for the Olink Target 96 Inflammation panel consisting of 92 immune‐related proteins. Seahorse Xfe96 Analyzer (Agilent) was used to assay metabolic flux of joint SFs (*n* = 24) seeded at 30 000 cells per well with and without 24 h TNFα stimulation at 10 ng/ml as previously described.^25^ Cell doubling events were calculated using trypan blue staining and Countess Automated Cell Counter (Invitrogen).

### 2.3 RNA sequencing analysis

Total RNA was extracted from isolated hand, hip, knee and foot OA SF (*n* = 24), from independent patients undergoing arthroscopy at a single anatomical joint, using RNeasy Mini Kit (Qiagen) and DNase treated (Qiagen DNase kit). Library preparation and RNA‐sequencing was performed by Genomics Facility at University of Birmingham using QuantSeq 3′ kit (Lexogen) and sequenced on Illumina's NextSeq 500. The sequence reads quality checks were carried out using fastQC, following which the bbduk from BBMap (version 38.87) software was used to trim Illumina adapters and polyA tails. Reads were mapped to hg38 reference human genome using Star Aligner. The R package DESeq2 was used to normalize raw read counts and perform statistical comparisons using VST transformations. Sequence data are available through the GEO database under accession number GSE219027.

### 2.4 3′‐end single‐cell RNA sequencing and raw data processing

Chromium 10X, scRNAseq analysis (Genomics Facility, University of Birmingham) was performed on a total of ∼2485 SF pooled from *n* = 4 obese and *n* = 4 normal‐weight OA patients, representing ∼310 cells/patient. Single cell 3′ cDNA libraries were constructed and sequenced using the Nextseq 500 (Illumina) platform. Libraries were pooled together and sequenced across four lanes, at an average read depth of ∼60 000 reads/cell. CellRanger version 3.0.1 was used to demultiplex the raw data and map to the hg38 reference genome (GRCh38.93). Sequence data are available through the GEO database (accession: GSE152815). 10x Genomic data sequencing metrics are detailed in Supplement Materials (SM. 4).

### 2.5 Data processing and analysis

Sequencing data were quality controlled and analysed using Seurat version 2.3.4 software (https://satijalab.org/seurat/). Datasets were merged, and low quality cells or doublets were filtered by excluding cells that expressed >6100 genes/cell and >20% mitochondrial gene expression. Technical noise was accounted for by scaling data based on nUMI and percentage of mitochondrial genes. Two thousand four hundred forty‐three cells remained post QC filtration. Lineage marker analysis as detailed by Mizoguchi et al., 2018 was used to confirm cells were strictly a homogenous fibroblast population.^22^ Data were subjected to global‐scale normalization and log‐transformed. Principal component analysis performed based on the 625 highly variable genes identified in the dataset and the most significant principal components (top 15 PCs) used for downstream t‐Distributed Stochastic Neighbour Embedding (t‐SNE) analysis to cluster the cells, with a resolution of .6.

### 2.6 Pseudotime analysis

Monocle version 2.9.0 was used to analyse pseudotime trajectories as detailed on monocle's tutorial page (http://monocle‐bio.sourceforge.net/). Following data normalization and variance estimation, genes used for pseudotime ordering were selected based on differentially expressed genes using ‘differentialGeneTest’ and filtering for genes expressed by at least 10 cells and with a q‐value of <.01. Using the DDRTree method, the dimensionality of the data was reduced, and cells were ordered in pseudo‐time. Full analysis script is detailed in Supplementary Materials (SM. 6).

### 2.7 Bioinformatic pathway analysis

DEG from each SF cluster (±1.5‐fold change, *p* < .05) was analysed using Ingenuity Pathway Analysis (IPA) (www.ingenuity.com) software. Core functional analysis was performed to identify canonical pathways and predicted upstream regulators significantly associated with DEG in each cluster subset. Fisher's exact test was used to calculate a *p*‐value of the association between DEGs, canonical pathways and upstream regulators (USRs).

### 2.8 Quantification of CHI3L1 and inhba by multiplex bead assay

Concentrations of CHI3L1 and Total Inhibin in cell supernatants (*n* = 8) were determined using multiplex technology (Luminex Screening Assay, R&D Systems). Multi‐plex analysis was performed according to the manufacturer's instructions and concentrations analysed using a Luminex 200 instrument (Luminex Corporation, Austin, Texas, USA).

### 2.9 Immunofluorescence and confocal microscopy

Tissues were embedded in Tissue‐Tek OCT medium and snap frozen in liquid nitrogen. For immunofluorescence, 10‐μm cryosections were rehydrated to water, postfixed with 4% PFA/PBS (Sigma‐Aldrich), blocked and permeabilized for 30 min in .25% Triton X‐100/5% normal donkey serum (NDS)/PBS (Vector Laboratories). Antibodies were diluted in 5% NDS/.1% Tween‐20/PBS. The following primary antibodies were incubated at 4°C overnight: anti‐FAPα (AF3715, R&D Systems), anti‐CXCL12 (MA5‐23759, Thermo Fisher Scientific), anti‐THBD (hpa002982, Atlas Antibodies), anti‐NPM1 (FC61991, Thermo Fisher Scientific), anti‐FBLN1(hpa001612, Atlas Antibodies), anti‐CD90 (AF2067, R&D Systems), anti‐c‐Myc (MAB36961, R&D Systems) and anti‐c‐Fos (PC05L, Merck). Staining was visualized with secondary antibodies (IgG‐NL493 (NL006) and IgG‐NL557 (NL010), 1:200, R&D Systems). Nuclei were visualized using ProLong Gold Antifade Mountant (P36931, Thermo Fisher Scientific). Stains were evaluated with a Leica DM6000 fluorescence microscope and using LAS AF Lite software.

### 2.10 Statistical analysis

Statistical analysis detailed in results and figure legends was performed using GraphPad Prism version 9 and SPSS version 27.

## 3 RESULTS

### 3.1 Load‐bearing and obesity differentially affect the secretory pro‐inflammatory phenotype of SFs

**FIGURE 1:**
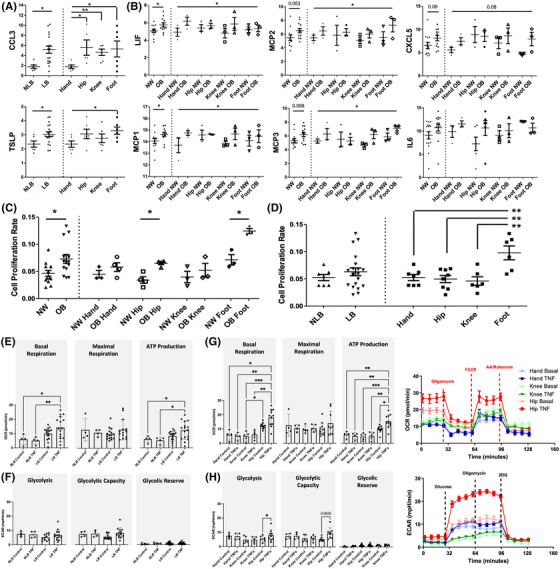
**Synovial fibroblast phenotyping by BMI and joint loading. (A and B)** Profiling of synovial fibroblast secretome using Olink Target 96 biomarker assay for inflammation. (**A)** Olink normalized protein expression (NPX) plotted for proteins of interest in load‐bearing (LB, *n* = 6), non‐load bearing (NLB, *n* = 18), hand (*n* = 6), hip (*n* = 6), knee (*n* = 6) and foot (*n* = 6) joints. (**B)** Olink NPX plotted for proteins of interest in normal ‐weight (NW, *n* = 12) and obese (OB, *n* = 12) joints, and BMI and joint (*n* = 3/group). (**C** and **D)** Cell doubling time plotted by BMI (*n* = 12/group), by BMI and joint (*n* = 3/group), by load‐bearing (LB, *n* = 6), non‐load bearing (NLB, *n* = 18), and by joint (*n* = 6/group) . (**E–H)** Seahorse analysis was used to measure oxygen consumption rate (OCR) and extracellular acidification rate (ECAR) of joint SF to determine mitochondrial respiration and glycolysis, respectively. (**E)** OCR of unstimulated control and TNFα treated (10 ng/ml for 24 hrs) SFs from load‐bearing (*n* = 16) and non‐load bearing (*n* = 4) joints from independent patients. (**F)** ECAR of unstimulated control and TNFα treated SFs from load‐bearing and non‐load bearing joints from independent patients. (**G)** OCR of unstimulated control and TNFα treated hand (*n* = 4), hip (*n* = 11) and knee (*n* = 5) SF, alongside graphical summary of glycolysis of SF from independent patients. (**H)** ECAR of unstimulated control and TNFα treated hand, hip and knee fibroblasts, alongside graphical summary of mitochondrial respiration. Two‐way ANOVA with Tukey HSD post‐hoc test used for seahorse assay analysis. Plotted are mean values ± SD. If not otherwise stated, Student's *t*‐test comparisons and ANOVA were performed between different joints, BMI, and loading from independent patients for Olink and proliferation data, plotted with mean values ± SEM. ***p < .001; **p < .01 and *p < .05.

Given our previous finding that the inflammatory phenotype of hip OA SFs was increased in patients who were obese,^16^ we were keen to expand these findings to other anatomical sites and to determine whether the effect of obesity was solely due to increased load‐bearing. To this end, the secretory profiles of SFs from both non‐load‐bearing (NLB, hand) and load‐bearing (LB, hip, knee, and foot) joints of both obese (OB, BMI > 30) and normal‐weight (NW, BMI 18.5–24.9) patients were assayed using Olink's Target 96‐marker inflammation panel (Figure 1A,B). In total, we identified 27 markers that were in range of the assay and further analysis revealed significant markers fell into three definitive categories, with differentially abundant markers being specific to load‐bearing joints, whilst others were regulated by obesity or specific to anatomical sites (Table 1).

**TABLE 1:** Summary of Olink Inflammation panel markers from heatmap. Average NPX data presented with standard deviation. Data analysed using unpaired students t‐test and one‐way ANOVA and Tukey posthoc test where *p < .05 and **p < .01.

	Non‐load bearing (*N* = 6)	Load bearing (*N* = 18)	NLB versus LB p‐value	Normal weight (*N* = 12)	Obese (*N* = 12)	NW versus OB p‐value	Hand (*N* = 6)	Hip (*N* = 6)	Knee (*N* = 6)	Foot (*N* = 6)	
CCL3	1.801 ± .3321	5.180 ± .7019	.0112 *	3.491 ± .8561	5.038 ± .8422	.2123	1.801 ± .3321	5.586 ± 1.525	4.657 ± .6301	5.365 ± 1.559	.0898
TSLP	2.347 ± .2244	3.044 ± .1714	.0437 *	2.879 ± .1928	2.861 ± .2427	.9553	2.347 ± .2244	3.069 ± .3338	2.763 ± .3065	3.300 ± .2554	.1303
LIF	5.548 ± .4038	5.131 ± .2307	.3520	5.080 ± .1991	5.762 ± .2291	.0351*	5.548 ± .4038	5.529 ± .2223	5.333 ± .4728	5.274 ± .2255	.9241
MCP1	14.22 ± .3835	14.38 ± .1563	.6408	14.05 ± .2203	14.63 ± .1656	.0486 *	14.22 ± .3835	14.60 ± .1879	14.26 ± .2959	14.28 ± .3342	.8060
MCP2	5.956 ± .3965	5.995 ± .3464	.9515	5.462 ± .3940	6.509 ± .3299	.0537	5.956 ± .3965	6.051 ± .7050	5.549 ± .3969	6.386 ± .7060	.7807
MCP3	5.767 ± .4506	5.766 ± .2821	.9990	5.322 ± .2994	6.211 ± .3242	.0563	5.767 ± .4506	5.381 ± .5346	5.433 ± .4655	6.484 ± .3931	.3347
CXCL5	6.503 ± .5987	7.616 ± .5775	.3084	6.552 ± .6697	8.124 ± .5797	.0899	6.503 ± .5987	8.718 ± .9305	7.836 ± .9893	6.295 ± .9776	.2071
IL6	10.70 ± .7286	10.78 ± .3944	.9237	10.59 ± .5190	10.92 ± .4552	.6380	10.70 ± .7286	11.43 ± .3354	9.557 ± .8497	11.34 ± .5531	.1826
CXCL1	11.34 ± .8805	11.85 ± .4130	.5656	11.67 ± .5554	12.02 ± .6392	.6832	11.34 ± .8805	13.04 ± .4983	12.25 ± .5914	10.26 ± .5679	.0388 hip versus foot*
CXCL6	9.843 ± 1.029	10.16 ± .4933	.7637	9.856 ± .5838	10.69 ± .5029	.2881	9.843 ± 1.029	11.60 ± .6238	10.28 ± .7468	9.385 ± .4486	.2102
IL8	10.75 ± .8738	11.46 ± .3752	.7063	10.94 ± .5800	11.71 ± .5662	.2013	10.75 ± .8738	12.49 ± .4275	11.55 ± .5515	10.33 ± .6942	.1346
SCF	2.910 ± .1469	2.850 ± .1153	.7848	2.827 ± .1157	2.903 ± .1491	.6926	2.910 ± .1469	2.671 ± .1357	2.740 ± .2901	3.139 ± .0873	.2958
uPA	9.184 ± .5934	8.799 ± .3927	.6190	8.563 ± .5159	9.035 ± .6126	.5643	9.184 ± .5934	9.500 ± .8672	9.311 ± .5466	7.587 ± .2783	.1319
OPG	11.70 ± .4450	12.11 ± .2642	.4343	11.93 ± .3356	12.09 ± .3145	.7249	11.70 ± .4450	12.98 ± .2423	11.03 ± .3944	12.33 ± .3243	.0069 hip versus knee **
MMP10	5.832 ± .3391	4.927 ± .4200	.2476	4.871 ± .4395	5.436 ± .5052	.4076	5.832 ± .3391	5.848 ± .9261	4.605 ± .7175	4.329 ± .4055	.2319
FGF21	2.872 ± .0737	2.840 ± .0613	.7794	2.778 ± .0785	2.917 ± .0543	.1598	2.872 ± .0737	2.993 ± .0732	2.669 ± .1143	2.857 ± .0965	.1243
MMP1	13.6 ± .4176	13.2 ± .4460	.6325	12.8 ± .5096	13.8 ± .4560	.1927	13.6 ± .4176	14.1 ± 1.027	13.2 ± .3609	12.3 ± .7281	.3400
CSF1	8.0 3 ± .3853	8.5 7 ± .2268	.2169	8.4 0 ± .2796	8.4 7 ± .2933	.9864	8.0 3 ± .3853	9.0 7 ± .4376	8.5 1 ± .4758	8.1 3 ± .1764	.2730
CCL20	5.3 4 ± .4904	4.9 1 ± .4107	.7803	4.6 9 ± .4105	5.4 4 ± .5086	.2548	5.3 4 ± .4904	5.8 1 ± .8207	4.5 7 ± .7649	4.2 4 ± .4205	.3101
IL18R1	2.8 1 ± .1421	2.9 8 ± .1900	.9148	2.8 1 ± .2102	2.9 7 ± .2095	.8008	2.8 1 ± .1421	3.2 2 ± .4369	2.8 6 ± .3285	2.6 6 ± .1601	.4833
ADA	3.0 6 ± .2380	2.7 8 ± .1675	.3497	2.8 6 ± .1741	2.7 5 ± .2251	.8329	3.0 6 ± .2380	2.7 2 ± .2741	2.6 1 ± .3277	2.7 2 ± .3209	.8315
TWEAK	3.3 2 ± .2771	3.2 1 ± .1748	.6898	3.2 3 ± .1678	3.2 4 ± .2466	.8713	3.3 2 ± .2771	3.3 5 ± .3350	3.4 4 ± .3762	2.8 3 ± .1435	.5224
IL7	2.1 3 ± .1803	2.2 2 ± .1376	.6288	2.1 6 ± .1344	2.2 3 ± .1812	.5214	2.1 3 ± .1803	2.4 7 ± .2998	2.2 9 ± .2613	1.9 9 ± .0867	.4094
Flt3L	3.9 7 ± .3398	4.1 1 ± .2343	.5191	4.0 9 ± .2353	3.9 8 ± .2569	.7537	3.9 7 ± .3398	4.3 3 ± .4266	4.1 8 ± .4159	3.7 6 ± .1682	.6834
TGFB‐1	3.2 2 ± .3213	3.6 7 ± .2310	.3370	3.5 1 ± .2130	3.6 5 ± .3286	.8525	3.2 2 ± .3213	4.0 1 ± .4139	3.5 0 ± .5722	3.5 0 ± .1326	.6204
HGF	3.9 0 ± .6175	3.5 6 ± .3100	.5253	3.8 6 ± .4186	3.4 3 ± .3656	.5098	3.9 0 ± .6175	3.4 7 ± .2806	3.7 1 ± .7047	3.4 9 ± .6258	.9005
DNER	1.0 2 ± .1463	.9 2 ± .0614	.5568	1.0 6 ± .0751	1.0 7 ± .0904	.9314	1.0 2 ± .1463	1.0 4 ± .1303	.9 3 ± .1230	.9 8 ± .0712	.8604

Cytokines influenced by loading included CCL3 (.01 *p*‐value) and TSLP (.04 *p*‐value), which overall were elevated in load‐bearing joints compared to the non‐loading‐bearing hand joint (Figure 1A). Cytokines influenced by obesity included LIF (.04 *p*‐value) and MCP1 (.04 *p*‐value), which were elevated in the secretome of obese SFs compared to normal weight SFs (Figure 1B). Additionally, a similar trend was observed in the secretory concentration of MCP2 (.054 *p*‐value) and MCP3 (.056 *p*‐value) (Figure 1B). Lastly, significant differences in the secretory concentrations of specific cytokines were identified upon comparing SFs from different anatomical joint sites, irrespective of obesity or load‐bearing, with SFs from the hip joint exhibiting the most inflammatory secretome. For example, CXCL1 (*p* < .04) was highest in the secretome from hip SFs, being significantly greater than in foot SF secretome and similar trends were observed with IL‐8 and CXCL6 (Table 1). Similarly, hip SFs also contained the greatest amount of OPG, being significantly greater than in the secretome of knee (*p* < .007) and to a lesser extent, that of hand SFs (Table 1). Together these data show SFs from various anatomical sites were differentially impacted by obesity and loading resulting in elevated secretion of specific pro‐inflammatory cytokine profiles compared to normal‐weight and non‐loading joints, respectively.

### 3.2 The proliferative activity of SFs is increased in obese patients but is dependent on anatomical site

In arthritic conditions, SFs adopt a hyperplastic phenotype contributing to inflammation and joint destruction.^26^ As such, we investigated whether anatomical location, obesity and load‐bearing played an integral role in influencing this proliferative phenotype. Comparing SFs isolated from obese and normal‐weight patients irrespective of anatomical location, obese SFs proliferated at a significantly greater rate than normal‐weight SFs (7.3 × 10^−2^ ± .008 vs. 4.7 × 10^−2^ ± .005, *p* < .01). The effect of obesity on proliferative rate was in general more pronounced in load‐bearing joints, with the greatest effect of obesity observed in foot (OB: 1.74 ± .066 vs. NW: 1.00 ± .13 vs., .008 *p*‐value) and hip joints (OB: 1.90 ± .08 vs. NW: 1.00 ± .18 vs., .003 *p*‐value) (Figure 1C). However, comparing SFs isolated from non‐loading joints (i.e., hands) and load‐bearing joints (i.e., hips, knees, or foot) irrespective of patient's BMI, we observed no overall effect of load‐bearing on SF proliferative activity (NLB joints: 5.2 × 10^−2^ ±.006 vs. LB joints: 6.3 × 10^−2^ ± .007, .4 *p*‐value) (Figure 1D). Lastly, we observed differences in the proliferative activity of SFs from different anatomical joints, with fibroblasts from hand (5.2 × 10^−2^ ± .02), hip (5.0 × 10^−2^ ± .02) and knee (5.0 × 10^−2^ ± .02) proliferating at a similar rate, but those from the foot (9.8 × 10^−2^ ± .03, .0007 *p*‐value) proliferating at a significantly greater rate (Figure 1D) suggesting loading may be a contributor. Overall, we find the hyperplastic phenotype of SFs is severely impacted by obesity resulting in increased proliferation especially in the hip and foot joint.

### 3.3 SF metabolic phenotype differs between anatomical sites and load‐bearing joints

Obesity results in significant metabolic complications that contribute to chronic inflammation and our most recent findings show the metabolic phenotype of hip OA SFs is significantly influenced by obesity.^25^ Here, we expanded this observation to different anatomical sites and investigated the effects of obesity and joint loading on the metabolic prolife of SFs from different joints and how they respond to inflammatory TNFα stimuli. We used seahorse analysis to measure oxygen consumption rate and extracellular acidification rate of joint SF to determine mitochondrial respiration and glycolysis, respectively. Interestingly, the most significant observations were driven by joint loading and anatomical location. Mitochondrial basal respiration and Adenosine 5'‐triphosphate (ATP) production was significantly altered in load‐bearing joints compared to non‐load bearing joints upon stimulation with TNFα, whilst no changes were observed in glycolysis (Figure 1E,F). Further observation of differences in SF metabolic phenotype between different joints found that basal respiration was elevated in hip SFs (12.2 ± 2.0), compared to either hand (5.9 ± 3.6) or knee SFs (4.9 ± 3.1), which indicates that SFs from specific joints have differing energy requirements (Figure 1G). TNFα stimulation elevated basal respiration levels in hip joint (17.9 ± 5.9) suggesting hip SFs are more responsive to inflammatory stimuli compared to hand (4.9 ± 2.5) and knee (6.1 ± 2.9) joint SF (Figure 1G). Similarly, ATP production was particularly elevated in hip SFs (9.9 ± 2.3) and further exasperated by TNFα stimulation (15.4 ± 4.6) suggesting higher energy demands in hip joint SFs compared to fibroblasts from other joint types (4.7 ± 2.5 knee, 6.0 ± 2.3 hand) (Figure 1G). No significant differences were noticed in basal levels of glycolysis between joints (4.9 ± 2.4 knee, 7.5 ± 1.9 hand, 5.5 ± 2.1 hip) (Figure 1G,H). However, upon inflammatory challenge with TNFa, hip SFs exhibited significantly greater induction in glycolysis from basal (8.0 ± 3.9, .03 *p*‐value) and greater glycolytic capacity suggesting hip SFs are metabolically more receptive to pro‐inflammatory stimulus than SFs from other joints (7.2 ± 2.3 hand, 4.8 ± 2.1 knee) (Figure 1H). No significant observations of note were identified with respect to the overall effect of obesity across all joints (Figure S1). These findings show SFs from loading joints are sensitive to pro‐inflammatory stimuli resulting in elevated mitochondrial respiration, largely driven by hip SFs, which also have increased glycolysis and ATP production, suggesting hip OA SFs have higher energy demands.

### 3.4 Obesity differentially affects the transcriptomic phenotype of SFs from both load‐bearing and non‐load‐bearing joints

**FIGURE 2:**
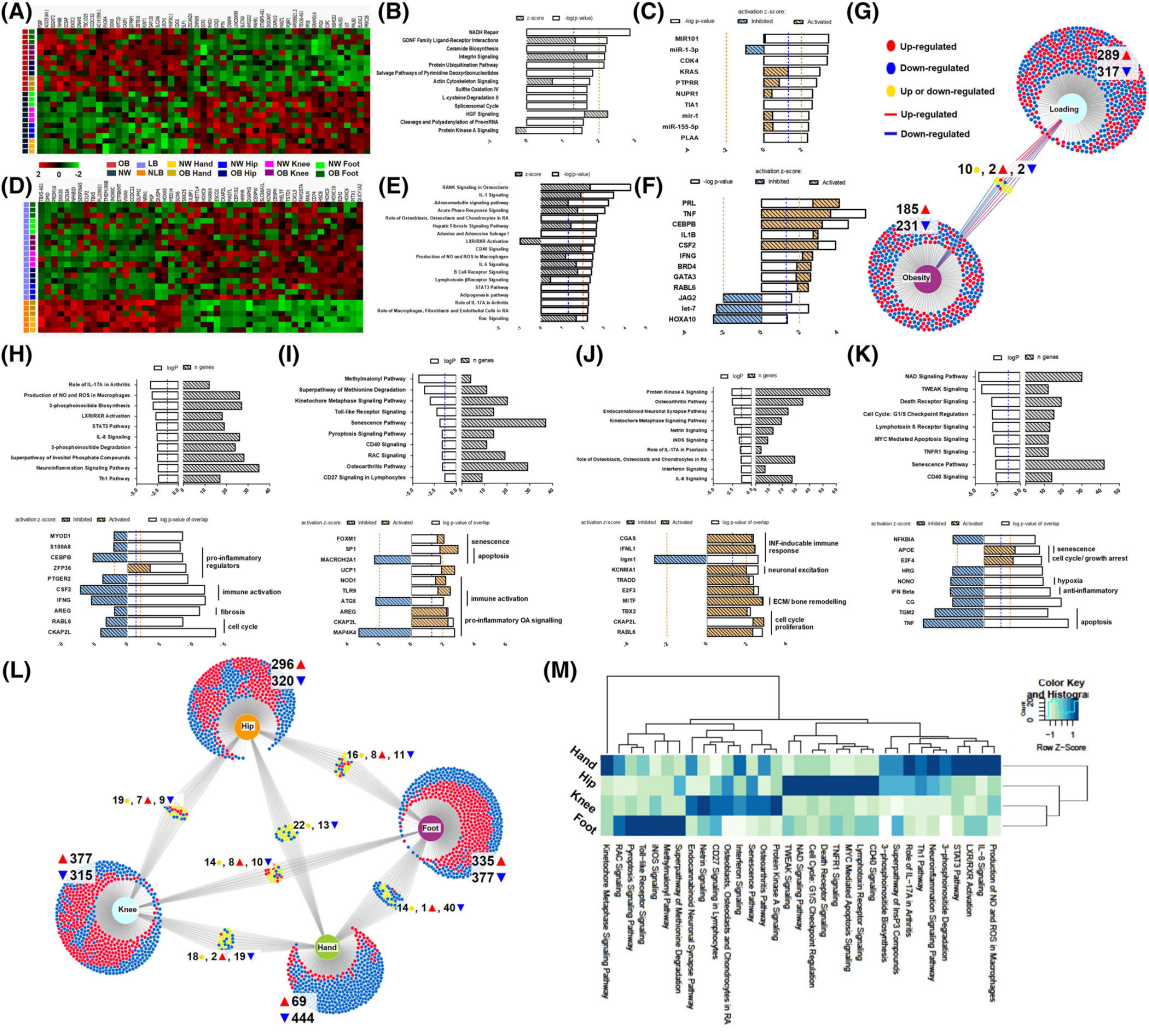
**Effects of obesity on synovial fibroblasts from different anatomical locations. (A and D)** Heatmap of DEGs in normal weight (NW, *n* = 12) versus obese (OB, *n* = 12), and load‐bearing (LB, *n* = 18) versus non‐load bearing (NLB, *n* = 6). (**B, E, H–K)** Most significantly enriched canonical pathways associated with DEGs in OB versus NW, in LB versus NLB and in OB versus NW hand, hip, knee and foot joints, respectively. Orange dash line denotes IPA z‐score and blue dash line denotes *p*‐value significance threshold of .05. (**C, F, H–K)** Top enriched upstream regulators associated with DEGs in OB versus NW, in LB versus NLB and in OB versus NW hand, hip, knee and foot joints, respectively. Orange dash line denotes IPA predicted activation score and blue dash line denotes *p*‐value significance threshold of .05. (**G)** Graphical venn diagram illustrates the number of significant DEGs identified in obese and load‐bearing joints and the number of DEGs, which are shared between these two analyses. (**L)** Graphical venn diagram illustrates the number of significant DEGs identified in obese joints at different anatomical locations and the number of DEGs which are shared between these joints. (**M)** Heatmap comparing top canonical pathways enriched in obese patients for each joint depicted by p‐value of enrichment. DEGs identified ±1.5‐fold change, *p* < .05.

Having identified differences in the inflammatory, proliferative and metabolic functional phenotype of fibroblasts associated with either load‐bearing, obesity or anatomical site, we next determined the key transcriptomic differences underpinning these findings. RNA‐sequencing analysis found distinct expression signatures exclusively regulated by either obesity or loading, with DEGs confirmed by RT‐qPCR analysis (see Supplementary Materials—SM. 3). Comparing SFs from obese patients with those from normal‐weight patients irrespective of joint site, a total of 416 DEGs (defined as ±1.5‐fold change, *p* < .05) were identified (Figure 2A, Table S1) which IPA functional annotations revealed to be involved in several metabolic pathways including ‘NADH repair’, ‘ceramide biosynthesis’, ‘protein ubiquitination’ and ‘pyrimidine salvage’ pathways (Figure 2B). Using IPA Upstream Regulator Analysis, KRAS, PTPRR and NUPR1 were identified as USRs of the observed obese SF DEG dataset (Figure 2C).

Conversely, upon comparing SFs from the non‐load bearing hand joints with SFs from all load‐bearing joints (i.e., knee, hip, foot) irrespective of patient BMI, a total of 606 DEGs were identified (Figure 2D, Table S2), with IPA functional annotations of these DEGs revealing several key inflammatory pathways including ‘RANK’, ‘IL‐1’ and ‘IL‐6’, together with the USRs TNF, CEBPB, IL1B and IFNG (Figure 2E,F). Notably, we found only 14 DEGs were common to both obesity and joint‐loading, of which only 4 genes had similar patterns of expression in both conditions (Figure 2G).

Further to these findings, analysis of the effects of obesity on individual joints established the profound effect of obesity on the SF transcriptome phenotype, even in non‐load bearing hand joints, with joint specific DEGs validated by RT‐qPCR (see Supplementary Materials—SM. 3). Specifically, 513 obesity DEGs were identified in hand OA SFs (Figure 2H, Table S3), 616 DEGs in the hip (Figure 2I, Table S4), 692 DEGs in the knee (Figure 2J, Table S5) and 672 DEGs in the foot (Figure 2K, Table S6) when comparing cells from obese and normal‐weight patients (Supplementary Tables S3‐S6). However, there was little overlap between the identified obesity‐associated DEGs in different joints even between the different load‐bearing joints, indicating that the effect of obesity on the SF transcriptome is joint type specific irrespective of loading (Figure 2L,M).

Conducting IPA on the obesity‐associated DEGs identified in each individual joint revealed the most significant canonical pathways and USRs involved in mediating the obese SF phenotype within each joint type. In hand joints, the top canonicals pathways included ‘phosphoinositide biosynthesis and degradation’ as well as ‘Th1’, ‘IL‐8’ and ‘STAT3’ signalling, which surprisingly had negative z‐scores, indicating pathway inhibition in the obese cells (Figure 2H). This was further supported by predicted inhibition of USRs involved in inflammation, immune activation and fibrosis (including CEBPB, CSF2, IFNG, AREG), and the significant downregulation in the expression of MMP9, S100A8, TYROBP and ARG2 in obese hand joint SFs compared to NW hand SFs (Figure 2H, Table S3). In the hip joint, IPA analysis of the obesity‐associated DEGs revealed significant canonical pathways involved in immune signalling pathways ‘CD27 and CD40 signaling’ as well as ‘osteoarthritis’ and ‘senescence’ pathways (Figure 2I). These pathways were in line with the up‐regulated expression of IKBKE, PALB2, UQCC3, COL4A4 and PTGS1 in the obese cells, which further correlated with activated USRs (FOXM1, SP1, TLR9, AREG and CKAP2L) involved in senescence and pro‐inflammatory OA signaling pathways (Figure 2I, Table S4). In the knee joint, the identified obese DEGs were significantly aligned to several inflammatory signaling pathways including ‘IL‐8 signaling’ and ‘interferon signaling’ (Figure 2J) which were concomitant with up‐regulated CXCL9, UBE2C and ADAM17 expression and the predicted activation of the USRs CGAS and IFNL1 (Figure 2J, Table S5). Finally, in the foot joint the identified obesity‐associated DEGs were significantly aligned to the canonical pathways ‘CD40 signaling’, ‘senescence’ and several ‘apoptosis’ signaling pathways (Figure 2K) with predicted activated USRs of senescence, cell cycle/ growth arrest (E2F4) and inhibited USRs of apoptosis (TGM2, TNF and IFNG). (Figure 2K). Comparing and contrasting the functional effects of obesity on SFs from different anatomical joints suggested the regulatory effects of obesity differed between joints. Dysregulation of inflammatory signaling pathways was a feature of both obese knee and hand SFs (Figure 2H,J,M), with dysregulation of both ‘IL‐8’ and ‘IL‐17A’ canonical signaling pathways and in both cases the identification of pro‐inflammatory USRs, albeit these USRs were predicted to be activated in the knee and inhibited in the hand resulting in in these pathways being positively enriched in the hand and negatively enriched in the knee (Figure 2H,J,M). Hip, knee and foot joints shared USRs or pathways of senescence, including canonical signaling pathways ‘CD40 signaling’ and ‘senescence pathway’ (Figure 2M), whilst hip and knee joints largely differed in other canonical pathways and USR (Figure 2I,J,M). Together these data indicate that obesity and loading drive very specific transcriptomic phenotypes in SFs with little commonality and finds that the inflammatory effect of obesity on the SF transcriptome is specific to each joint irrespective of loading.

### 3.5 Obesity is associated with distinct SF subsets in hip OA

**FIGURE 3:**
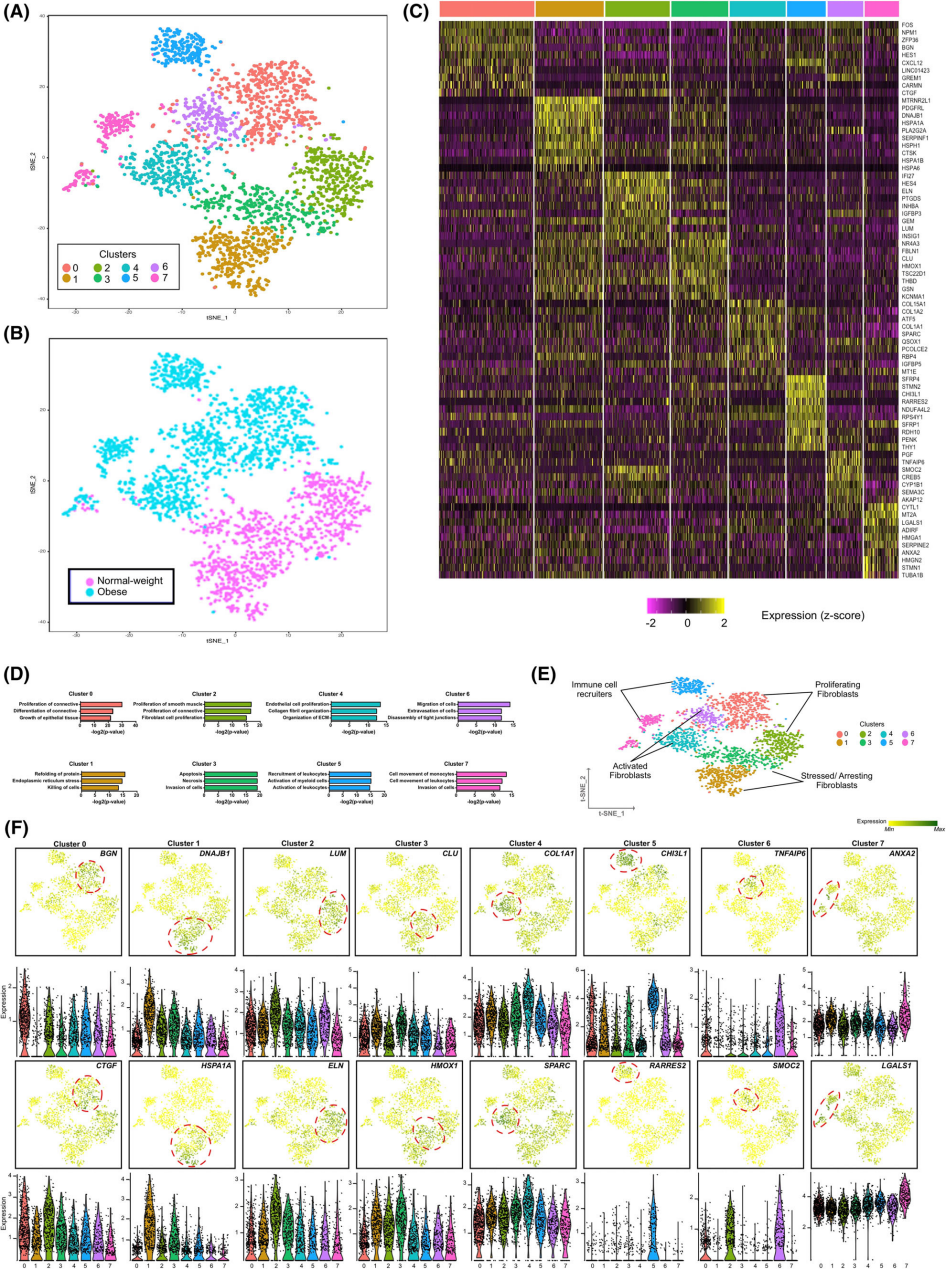
**Single cell RNA‐sequencing identifies distinct synovial fibroblasts subsets in obese and normal‐weight OA patients. (A)** t‐SNE analysis of SF scRNAseq data showing eight fibroblast subsets. In total, scRNAseq was performed on 2485 SF from *n* = 4 normal‐weight OA patients and *n* = 4 obese OA patients. (**B)** t‐SNE plot showing the separation between fibroblasts from normal‐weight OA patients and fibroblasts from obese OA patients. (**C)** Heatmap showing the z‐score average gene signature expression of the top 10 most differentially expressed genes within each of the 8 SF clusters. (**D)** Significant biological processes for each cluster. Differentially expressed genes were analysed using IPA software. The significance of the association of a given disease function with the genes within a given subset was measured in two ways. Firstly, by the ratio of the number of differentially expressed genes in the dataset that mapped to the diseases/function divided by the total number of genes associated with that function. Secondly, Fisher's exact test was used to calculate a *p*‐value of the association between the genes and the disease function. (**E)** Clusters were assigned functional endotypes based on the canonical pathways identified in the IPA analysis. (**F)** FeaturePlots displaying expression of cluster specific markers on the t‐SNE map along with violin plots showing the expression levels (y‐axis) of these markers for each cluster (x‐axis). Additional enriched genes are detailed in Figures S2–S9.

Collectively, our observations found the most profound effects of obesity on the SF phenotype were in the hip joint, with effects on the inflammatory, proliferative and metabolic phenotype. As such we next investigated whether these obesity‐associated effects could be attributed to heterogeneity in particular SF subsets using scRNAseq. This analysis revealed eight fibroblast subsets defined by specific transcriptomic profiles (Figure 3A,B), with clusters 0, 4, 5, 6 and 7 present in obese hip OA patients, and clusters 1, 2 and 3 found in normal‐weight OA patients. The fibroblast compositions of these clusters included 510 cells in cluster 0 (98.6% OB; 1.4% NW), 369 cells in cluster 1 (O% OB; 100% NW), 357 cells in cluster 2 (3.9% OB; 96.1% NW), 307 cells in cluster 3 (.7% OB; 99.3% NW), 306 cells in cluster 4 (95.4% OB; 4.6% NW), 211 cells in cluster 5 (99.1% OB; .9% NW), 195 cells in cluster 6 (99.5% OB; .5% NW) and 188 cells in cluster 7 (87.2% OB; 12.8% NW), see Supplementary Materials—SM. 5. The cells in each cluster were defined by distinct gene expression patterns which define each population, as seen in the expression heatmap of the top 10 genes in each cluster (Figure 3C) and detailed in Tables S7 and S8.

**FIGURE 4:**
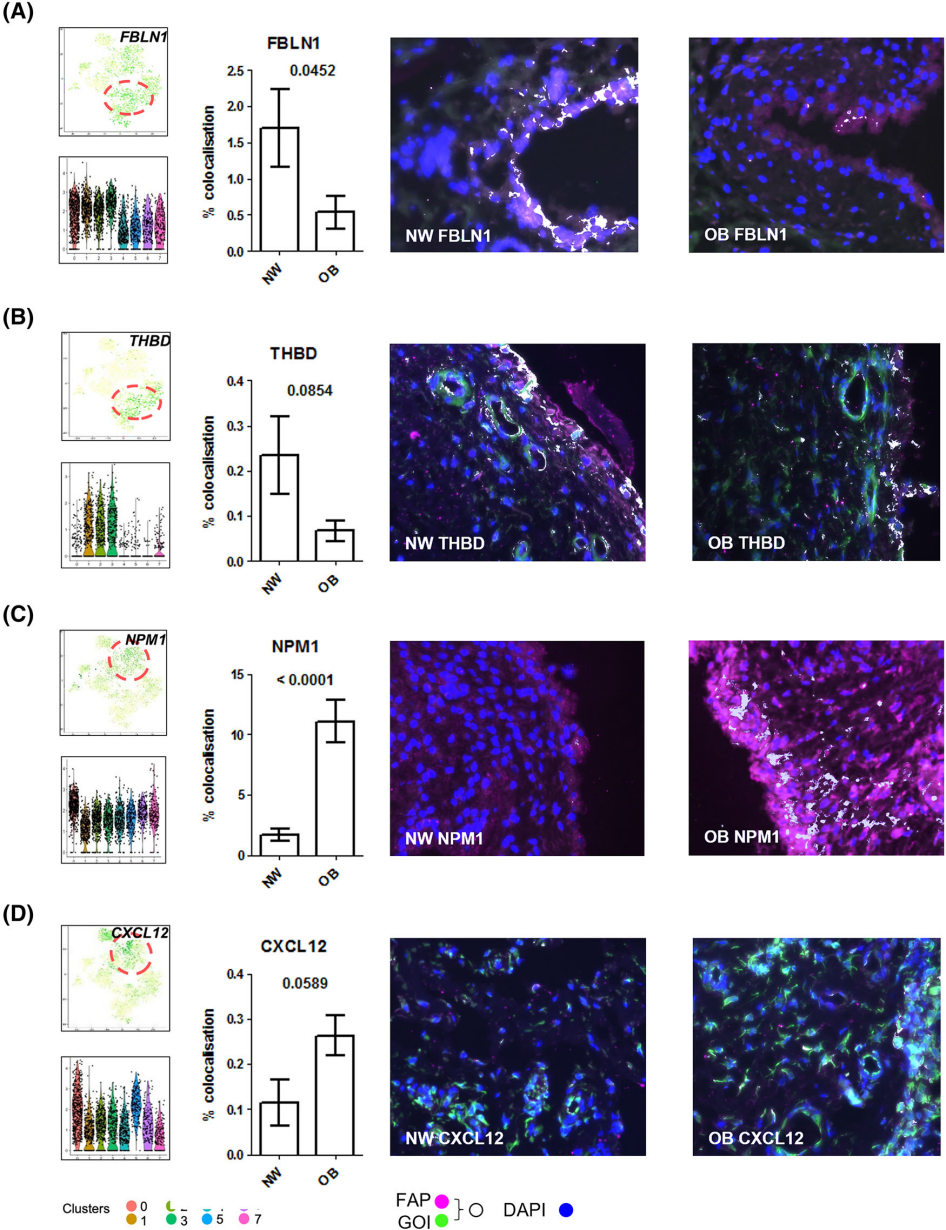
**Distinct synovial fibroblast clusters are differentially distributed in synovium tissue of obese and normal weight patients**. Differences in single cell RNA‐sequencing cluster distribution between obese and normal weight patients were validated in OA hip synovium tissue. (**A–D)** Panels consist of FeaturePlots displaying cluster specific markers on the t‐SNE map along with violin plots showing the expression levels (y‐axis) of these markers for each cluster (x‐axis), bar graph representing quantification of colocalized genes of interest (GOI) with FAP labelled fibroblasts and representative immunofluorescence images in OA hip synovial tissue from normal weight (NW) and obese (OB) patients. Cluster markers of interest (GOIs) were visualized with (**A)** FBLN1, (**B)** THDB, (**C)** NPM1 and (**D)** CXCL12 (green), synovial fibroblasts were visualized with FAP (pink), and nuclei were stained with DAPI (blue), colocalization has been pseudo‐coloured (white). IF colocalization analysis. *N* = 8 plotted with mean ± SEM and analysed by student's *t*‐test.

We next examined the identified DEGs (±1.5 fold change, *p* < .05) within each cluster using IPA software. Core functional analyses identified canonical pathways associated with the DEGs that functionally defined the unique transcriptomic profile of each cluster (Figure 3D, summarized in Figures S2–S9 highlighting the top 10 DEGs). Thus, the clusters were functionally assigned into four endotypes, namely ‘activated fibroblasts’, ‘stressed/arresting fibroblasts’, ‘proliferating fibroblasts’ and ‘immune cell recruiters’ based on the canonical pathways identified (Figure 3E). Notably, ‘activated fibroblasts’ (clusters 4 and 6) and ‘immune cell recruiters’ (clusters 5 and 7) endotypes described transcriptome profiles that were predominant in obese OA clusters. In contrast, the ‘stressed/arresting’ fibroblast endotypes (cluster 1 and 3) almost entirely represented normal‐weight fibroblasts. The ‘proliferating fibroblast’ endotype was present in fibroblasts from both obese and normal‐weight OA patients, although these were defined by distinctive DEG profiles (Figure 3D,E). t‐SNE and violin plots featuring gene expression (Figure 3F) showed that ‘activated fibroblasts’ had gene expression signatures associated with the production, maintenance and rearrangement of the extracellular matrix including collagens (COL1A1, COL1A2), the collagen‐binding matrix protein osteonectin (secreted protein acidic and rich in cysteine [SPARC]), tumor necrosis factor‐inducible gene 6 protein (TNFAIP6) and osteonectin‐related protein (SMOC2) (Figure 3F, Figures S2 and S3). The ‘immune cell recruiters’ subset consisted of genes associated with the activation and recruitment of leukocytes, neutrophils and monocytes, with high expression of genes that mediate inflammation and innate immune responses including Chemerin (RARRES2), Chitinase3‐like 1 (CHI3L1), SFRP1, Thy1 (CD90), SFRP4 and Galectin‐1 (LGALS1) (Figure 3F, Figures S4 and S5). Normal‐weight fibroblasts were largely represented by gene signatures involved in apoptosis and cellular stress response, with high expression of SERPINF1, CLU, DNAJB1 and HSPA1A/HSPA1B (Figure 3F, Figures S6 and S7). Fibroblasts that were functionally defined as ‘proliferating’ were represented by both the gene signature of LUM, INHBA and ELN in the normal weight cluster 2 cells, and by the expression of BGN, CTGF and CXCL12 in obese cluster 0 cells (Figure 3F, Figures S8 and S9). Differences in cluster distribution between obese and normal weight patients were validated in synovium tissue using immunofluorescence. Normal weight cluster 3 markers Fibulin 1 and Thrombomodulin, encoded by FBLN1 and THBD, were co‐localised with SFs enriched in normal weight synovium tissue (Figure 4A,B). Whilst obese cluster 0 markers Nucleophosmin and Stromal cell‐derived factor 1, encoded by NPM1 and CXCL12, were colocalized with fibroblasts enriched in synovium tissue from obese patients (Figure 4C,D). Overall, distinct SF subpopulations were identified by scRNAseq which can be grouped into four endotypes, with obese SFs predominantly defined by pro‐inflammatory SFs with ‘activated’ and ‘immune recruiter’ endotypes.

### 3.6 Inflammatory SF subsets in obese patients spatially localize in sublining and lining layers of OA synovium and can be distinguished by differential expression of transcriptional regulators MYC and FOS

**FIGURE 5:**
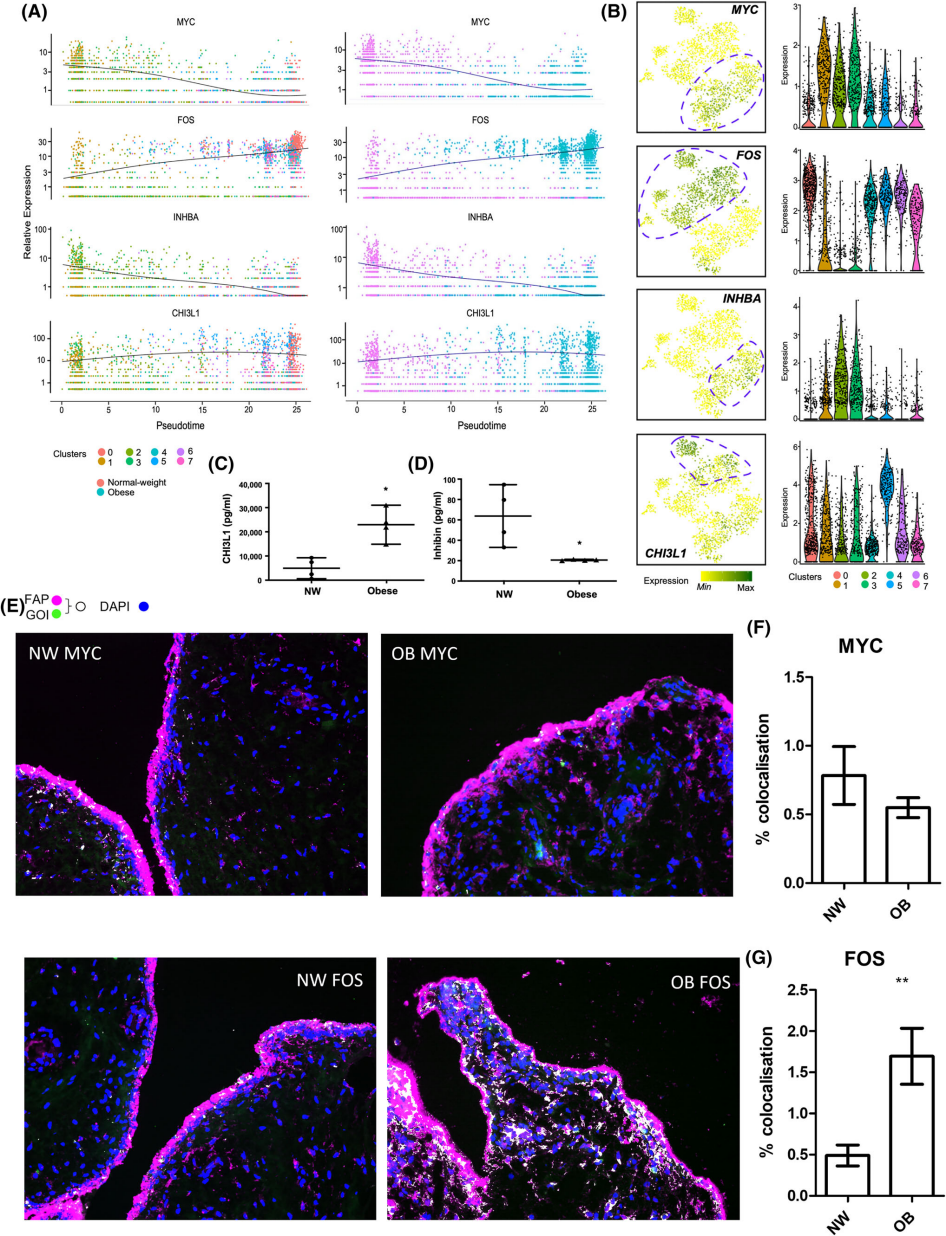
**Expression dynamics of markers which distinguish normal weight and obese patient synovial fibroblasts. (A)** Expression of transcriptional regulators MYC and FOS, and secretory factors INHBA and CHI3L1 along the pseudotime axis, overlaid with representation of clusters (left) and sample distribution (right) using Monocle pseudotime trajectory. Cells are ordered in pseudotime based on differentially expressed genes (*q*‐value < .01). (**B)** FeaturePlots displaying cluster specific expression of MYC, FOS, INHBA and CHI3L1 on the t‐SNE map along with violin plots showing the expression levels (y‐axis) of these markers for each cluster (x‐axis). (**C and D)** Median concentrations of INHBA (inhibin) and CHI3L1 in 24‐h conditioned media from normal‐weight and obese OA SF by Luminex. Bars represent median concentration in pg/ml from n = 4 patients per cohort. **(E)** Representative immunofluorescence imaging for c‐Fos or c‐Myc (GOI, green) in OA hip synovial tissue from normal weight (NW) and obese (OB) patients. SF were visualized with FAP (pink), and nuclei were stained with DAPI (blue), colocalization of FAP and GOI has been pseudo‐coloured (white). See Figure S10 for additional panels. (F and G) Quantification of colocalized c‐Myc or c‐Fos with FAP labelled SF. *N* = 8. Means plotted ± SD and analysed by student's *t*‐test **p < .01.

Next, we performed pseudo temporal ordering on normal‐weight and obese fibroblasts to investigate the dynamics in changing expression patterns of DEGs identified in each cluster. Amongst these genes, we observed a striking transition in the expression of the transcriptional regulators MYC and FOS (Figure 5A). FOS was predominately expressed in obese clusters whilst MYC was predominantly expressed in normal‐weight clusters (Figure 5B). Similarly, pseudo temporal expression dynamics also identified transitional expression changes in INHBA in normal‐weight clusters, and CHI3L1 present in the obese clusters (Figure 5A,B). Since the gene products of INHBA (Inhibin) and CHI3L1 (Chitase3‐like 1) are secreted proteins, we examined whether this was reflected in SF conditioned media. In line with mRNA expression, SFs from obese patients secreted significantly greater amounts of Chitinase3‐Like‐1 (229.5 ng/ml, 95% CI = 123.7–335.3 vs. 49.5 ng/ml, 95% CI = −15.4–114.3, *p* < .05) and significantly lower amounts of Inhibin (20.61 pg/ml, 95% CI = 20.16–21.22 vs. 63.79 pg/ml, 95% CI = 18.82–108.7, *p* < .05), compared to SFs from normal‐weight patients (Figure 5C,D). Additionally, immunofluorescence labelling of c‐Myc and c‐Fos protein in synovial tissue from hip OA patients confirmed both the observed differences in fibroblast transcriptomic profiles in‐situ at the protein level as well as their spatial cellular localization (Figure 5E). c‐Myc staining appears more intense in normal‐weight OA synovial tissue than obese synovium, where there was minimal expression observed in the synovial sublining tissue, as supported by quantitative colocalization analysis, which showed a trend for increased Myc staining in normal‐weight OA, although not significant (*p* = .0924) (Figure 5F). Conversely, c‐Fos staining was significantly greater in obese OA synovial tissue (*p* = .0099), with expression in both the synovial sublining and in the synovial lining layer where it was co‐localized three times more with fibroblast activation protein (FAP)‐positive SFs in obese tissue (Figure 5G). By comparison c‐Fos expression in normal‐weight synovium was largely confined to the lining layer only (Figure 5E). Taken together, these data suggest that pro‐inflammatory SFs in obese patients localize in the sublining and lining layers of OA synovium tissue which can be distinguished by c‐Fos whilst ‘stressed/ arresting’ SFs in normal weight patients are only present in the lining layer and distinguished by c‐Myc.

## 4 DISCUSSION

OA is a multifaceted condition, which poses a significant challenge for the successful clinical development of therapeutics due to its heterogeneity. However, understanding and classifying the molecular phenotypes, also known as endotypes, of OA pathogenesis could provide invaluable phenotype‐directed routes for stratifying subgroups of patients for targeted therapeutics, leading to greater chances of success in trials. Our previous work identified obesity as a critical factor in the heterogeneity of synovial joint inflammation in hip OA patients.^16^ As such, this study aimed to expand these findings to both load‐bearing and non‐load bearing joints to further investigate the intersectional effects of obesity and loading on OA synovial pathogenesis and to identify key molecular endotypes that could stratify patients for targeted therapeutics. Our findings demonstrate that the dynamic inflammatory landscape of OA SFs is significantly impacted by joint loading and obesity, as well as being influenced by anatomical site. Additionally, we address the heterogeneity in obese and normal‐weight OA patients by identifying distinct differences in SF subsets, which can be characterised into four functional molecular endotypes. We find that the presence and predominance of these endotypes is distinctly different in obese hip OA patients compared to normal‐weight patients. As such, this study highlights the disease heterogeneity across OA joints and provides a compelling rationale for the necessity of determining robust molecular endotypes to effectively stratify patients for the best therapeutic outcomes.

Obesity is a known risk factor for the development of OA in both load bearing and non‐load bearing joints,^27^, ^28^ suggesting the resulting inflammatory phenotypes are not solely due to increased load as we report here. Here, we confirm that obesity affects the inflammatory phenotype of SFs even in non‐load bearing hand joints, with elevated secretion of MCP1, MCP2, MCP3 and LIF, and has a profound but anatomical‐site specific differential effect on their transcriptomic landscape. Elevated MCP‐1 in synovial fluid correlates with increased macrophage activation in joint tissue,^29^ whilst LIF facilitates sustained inflammatory fibroblast activation^30^ suggesting these markers are fundamental in sustaining an enhanced pro‐inflammatory phenotype in obese patients. This enhanced pro‐inflammatory phenotype is also evident in the hyperplastic nature of obese SFs, which we find have increased proliferation rates. Synovial hyperplasia further contributes to inflammation and joint destruction in arthritis and our findings here suggest obesity exacerbates these pathological effects regardless of the joint location. Recent studies have provided evidence that increases in RA fibroblast proliferative inflammatory activity are underpinned by alterations in the activity of key metabolic pathways.^31^, ^32^ However, despite previously finding that SFs from obese hip OA patients exhibit a different inflammatory metabotype,^25^ in this study there was no overall effect of obesity on the functional metabolic SF phenotype when examining across all joint types.

We also identified CCL3 and TSLP as cytokines that were more highly secreted from fibroblasts isolated from load‐bearing joints (knees, hips, foot) compared to the non‐load‐bearing hand joint but were not differentially secreted by obesity, which might be indicative of their induction in response to load‐induced cartilage damage. CCL3 is elevated in severe knee OA pathogenesis and cited as a biomarker for severity of joint destruction with respect to the degree of cartilage damage.^33^ Cartilage‐damage induces the release of DAMPs and activation of toll‐like receptors (TLR) known to induce TSLP, which is elevated in RA patients and believed to drive inflammatory arthritis.^34^, ^35^, ^36^, ^37^ Many of the pathways identified in our transcriptomic analysis also allude to the effects of pathological loading directly impacting joint inflammation and cartilage degradation further supporting these observations. Interestingly, loading only appears to affect the proliferative rate of fibroblasts in the most load‐bearing joint, that is, the foot joint, suggesting the effects of loading differs between anatomical locations. In addition to this, we also note several markers that distinguish specific anatomical sites, confirming a significant level of heterogeneity in the SF phenotype between joints. This is further supported by transcriptomic analysis where the effect of obesity on the OA fibroblast transcriptome is markedly different between SFs isolated from different anatomical sites.

We find the effect of obesity extends further to fibroblast subsets within the synovium, with distinct subset endotypes responsible for specific inflammatory phenotypes. These clusters draw similarity to fibroblast subsets reported in RA, suggesting OA pathogenesis in obese patients may be akin to the more inflammatory RA joint. Obese OA specific clusters are involved in the activation and recruitment of immune cells, which are reminiscent of ‘immune‐effector fibroblasts’ reported in RA known to regulate and recruit immune cells.^23^ Here, this subset expresses CHI3L1 a known autoantigen in RA,^38^ which is associated with several inflammation‐related disorders.^39^, ^40^, ^41^, ^42^ CHI3L1 mediates inflammation,^39^ cartilage degradation and remodeling^40^, ^43^ and regulates macrophage and T‐cell recruitment,^39^, ^43^ supporting the immune regulatory phenotype of this cluster. In contrast, normal‐weight SF populations are characterised as mediating apoptosis and necrosis, in keeping with a lower proliferative capacity.^16^ Necrotic cells trigger rapid inflammatory responses,^44^, ^45^ which activate SFs through TLRs and downstream pro‐inflammatory signaling pathways.^46^, ^47^ These normal‐weight OA fibroblast clusters also express INHBA, a component of activin known to rapidly transition apoptotic cells to secondary necrosis in the lungs.^48^ Lastly, our results suggest an obesity‐related regulatory switch involving two transcription factors, MYC and FOS, which may drive the distinctive endotypes we observe. Interestingly, these two transcriptional regulators can be used to distinguish spatial localization of SFs described by endotypes. The immune regulatory inflammatory SFs from obese patients appear to localize in the both sublining and lining layers of synovium tissue based on c‐Fos staining, reminiscent of inflammatory RA SFs described by Croft et al. 2019, whilst subsets identified in normal weight SFs, positive for c‐Myc, are mostly confined to the lining layer similar to previously reported OA fibroblasts, suggesting obese OA synovium is more akin to inflammatory RA synovium.^23^ MYC regulates SF proliferation, invasiveness,^49^ and apoptosis,^50^ in keeping with the apoptosis pathways established in normal‐weight SF clusters. Whilst many joint destructive cytokines and MMPs are transcriptionally regulated by FOS/Activator Protein‐1 (FOS/AP‐1)^51^ supporting the ‘activated’ and ‘immune‐cell recruiter’ fibroblast subsets identified in obese SF populations. Furthermore, a diet‐induced weight‐loss study also demonstrated a similarly interesting regulatory switch from NFKB1, an upstream regulator of FOS, to MYC/MAX following weight loss.^52^ These results further suggest that SFs from normal‐weight and obese OA patients utilize alternative pathways to mediate inflammation in the joint. Targeting these specific pathways within patient subgroups, defined by molecular and clinical endotypes, could pave the way for overall better disease management. Importantly, CHI3L1 and INBHA are secreted from obese and normal weight OASF, respectively. As such these could serve as patient stratification aids to identify those patients with distinctive molecular endotypes for more targeted therapeutics.

Collectively, these data emphasize the breadth, magnitude and differential effect of obesity and load‐bearing on the SF phenotype, resulting in heterogeneous inflammatory fibroblast endotypes, and therefore the potential of these factors to influence the inflammatory pathology of OA. This begs the question of what impact do these fundamental differences have on the likely efficacy of current anti‐inflammatory treatments and what are the implications for the development of new drugs, since targets identified within a particular subgroup may not be relevant across other joint types and patient groups. This also raises the question as to what extent do researchers need to interrogate SFs and other joint cell types from specific sites, particularly in early drug discovery studies, in order to identify appropriate and effective targets for the development of therapeutics. An interesting observation made in this study is the responsiveness of hip OA SFs, which compared to SFs from other joints, have higher energy demands and are more metabolically responsive to inflammatory stimuli. Such differences are also likely to impact the responsiveness of these joint cells to therapeutics. This is further complicated by the identification of multiple fibroblast clusters which differentiate normal‐weight and obese OA patient synovitis supporting a rationale to therapeutically target specific fibroblast subsets that are driving inflammatory versus proliferative phenotypes. Whilst this may border on personalized drug development, understanding the molecular endotypes that govern OA pathogenesis across subgroups will undoubtedly aid in the development of targeted therapies particularly when coupled with clinical endotypes. Such targeted developments have already been evidenced in asthma research with significant benefit.^5^

Lastly, it is important to consider the observations reported here in view of limitations associated with a relatively small sampling size. As such, the patients used here are only representative of a small fraction of the greater population of OA patients. Expanding such analysis to encompass a larger cohort of patients will not only power these observations but also refine the endotypes identified here. Also, for single cell analysis the SFs used were exclusively performed on the hip joint. Moreover, whilst the number of cells used for 10X is within the recommended range (100–8000 cells), this is on the lower end of the scale. As such, authors used SCOPIT statistical tool to ensure confidence in the data presented. Retrospective statistical modelling determine only 201 cells are required to detect subpopulations with a .95 probability, suggesting over 10 times the necessary number of cells were sequenced in this study which is greater than similar publications characterising SFs in RA.^22^, ^23^, ^53^

In conclusion, the findings of this study highlight the significance of obesity in both load‐bearing and non‐load bearing joints in changing the inflammatory molecular endotype OA SFs. Refining these SF endotypes in relation to clinical phenotypes may identify different patient subgroups with personalized targets for therapeutic intervention, thus providing a critical line of sight between drug targets and patient selection, facilitating optimal clinical trial design.

## CONFLICT OF INTEREST STATEMENT

The authors declare that they have no competing interests.

## CONSENT TO PARTICIPATE

Informed consent was obtained from all individual participants included in the study.

## Supporting information

SM.1 ‐ Patient Characteristics Summary
SM.2 – Unsupervised hierarchical clustering and PCA analysis of joint RNAseq data
SM.3 – RT‐qPCR validations of bulk RNA sequencing data
SM.4 ‐ 10X Genomics data Metrics
SM.5 ‐ 10X Genomics data Sample Distribution
SM.6 ‐ Analysis pipeline and script for 10X Genomics data
SM.6 Summary of analysis strategy for single cell RNA sequencing of OA hip‐isolated fibroblast samples.
FIGURE S1 Graphical summary of mitochondrial respiration and glycolysis of obese and normal weight OA joint synovial fibroblasts.
FIGURE S2 Summary of DEGs in Cluster 4.
FIGURE S3 Summary of DEGs in Cluster 6.
FIGURE S4 Summary of DEGs in Cluster 5.
FIGURE S5 Summary of DEGs in Cluster 7.
FIGURE S6 Summary of DEGs in Cluster 1.
FIGURE S7 Summary of DEGs in Cluster 3.
FIGURE S8 Summary of DEGs in Cluster 0.
FIGURE S9 Summary of DEGs in Cluster 2.
FIGURE S10 Pseudo‐coloured accessible IF panel.

Supporting Information
