# Supplementary material for: Obesity defined molecular endotypes in the synovium of patients with osteoarthritis provides a rationale for therapeutic targeting of fibroblast subsets
Source: Clin Transl Med. 2023 Apr 3;13(4):e1232. doi: 10.1002/ctm2.1232 (PMC10068310; doi:10.1002/ctm2.1232)
Supplement: Supplementary file 1 — SM.1 ‐ Patient Characteristics Summary SM.2 – Unsupervised hierarchical clustering and PCA analysis of joint RNAseq data SM.3 – RT‐qPCR validations of bulk RNA sequencing data SM.4 ‐ 10X Genomics data Metrics SM.5 ‐ 10X Genomics data Sample Distribution SM.6 ‐ Analysis pipeline and script for 10X Genomics data SM.6 Summary of analysis strategy for single cell RNA sequencing of OA hip‐isolated fibroblast samples. FIGURE S1 Graphical summary of mitochondrial respiration and glycolysis of obese and normal weight OA joint synovial fibroblasts. FIGURE S2 Summary of DEGs in Cluster 4. FIGURE S3 Summary of DEGs in Cluster 6. FIGURE S4 Summary of DEGs in Cluster 5. FIGURE S5 Summary of DEGs in Cluster 7. FIGURE S6 Summary of DEGs in Cluster 1. FIGURE S7 Summary of DEGs in Cluster 3. FIGURE S8 Summary of DEGs in Cluster 0. FIGURE S9 Summary of DEGs in Cluster 2. FIGURE S10 Pseudo‐coloured accessible IF panel. [file CTM2-13-e1232-s001.docx]

Supplementary Materials for

**Obesity defined molecular endotypes in the synovium of patients with osteoarthritis provides a rationale for therapeutic targeting of fibroblast subsets**

Susanne N. Wijesinghe, Amel Badoume, Dominika E. Nanus, Archana Sharma-Oates, Hussein Farah, Michelangelo Certo, Fawzeyah Alnajjar, Edward T. Davis, Claudio Mauro, Mark A. Lindsay, Simon W. Jones^*^

*Corresponding author.

Email: s.w.jones@bham.ac.uk

**This PDF file includes:**

SM.1 Patient Characteristics Summary

SM.2 Unsupervised hierarchical clustering and PCA analysis of joint RNAseq data

SM.3 RT-qPCR validations of bulk RNA sequencing data

SM.4 10X Genomics data Metrics

SM.5 10X Genomics data Sample Distribution

SM.6 Analysis pipeline and script for 10X Genomics data

Supplementary Figures - S1 to S10

**Other Supplementary Materials for this manuscript include the following:**

Supplementary Tables

Table S1 – Obesity DEGs

Table S2 – Loading DEGs

Table S3 – Hand DEGs in obesity

Table S4 – Hip DEGs in obesity

Table S5 – Knee DEGs in obesity

Table S6 – Foot DEGs in obesity

Table S7 – Top 10 single cell DEGs per cluster

Table S8 – All single cell DEGs for all clusters

SM.1 - Patient Characteristics Summary

|  | **Normal Weight**  **(BMI 18.5- 24.9)** | **Obese**  **(BMI> 30)** | **Non-Load bearing**  **(Hand)** | **Load bearing**  **(Hip, Knee, Foot)** |
| --- | --- | --- | --- | --- |
| **Number of patients** | 12 | 12 | 6 | 18 |
| **Age (years)** | 65.2 ± 9.1 | 63.9 ± 8.0 | 58.2 ± 5.0 | 66.7 ± 8.3 |
| **Sex (% female)** | 66.7 ± 4.9 | 66.7 ± 4.9 | 66.7 ± 5.2 | 66.7 ± 4.9 |
| **BMI (kg/m^2^)** | 23.2 ± 1.8 | 33.8 ± 2.0 | 28.1 ± 5.8 | 28.6 ± 5.8 |
| **Waist Circumference (cm)** | 82.5 ± 9.8 | 110.0 ± 11.5 | 97.8 ± 15.7 | 95.7 ± 18.4 |
| **Hip Circumference (cm)** | 95 ± 13.3 | 121.7 ± 11.7 | 112.4 ± 13.6 | 107.0 ± 19.8 |
| **Waist:Hip Ratio** | 0.88 ± 0.14 | 0.91 ± 0.09 | 0.87 ± 0.09 | 0.90 ± 0.13 |
| **Diabetes (n )** | 0 | 1 | 0 | 1 |
| **Hypertension (n)** | 1 | 4 | 1 | 4 |

SM.2 – Unsupervised hierarchical clustering and PCA analysis of joint RNAseq data


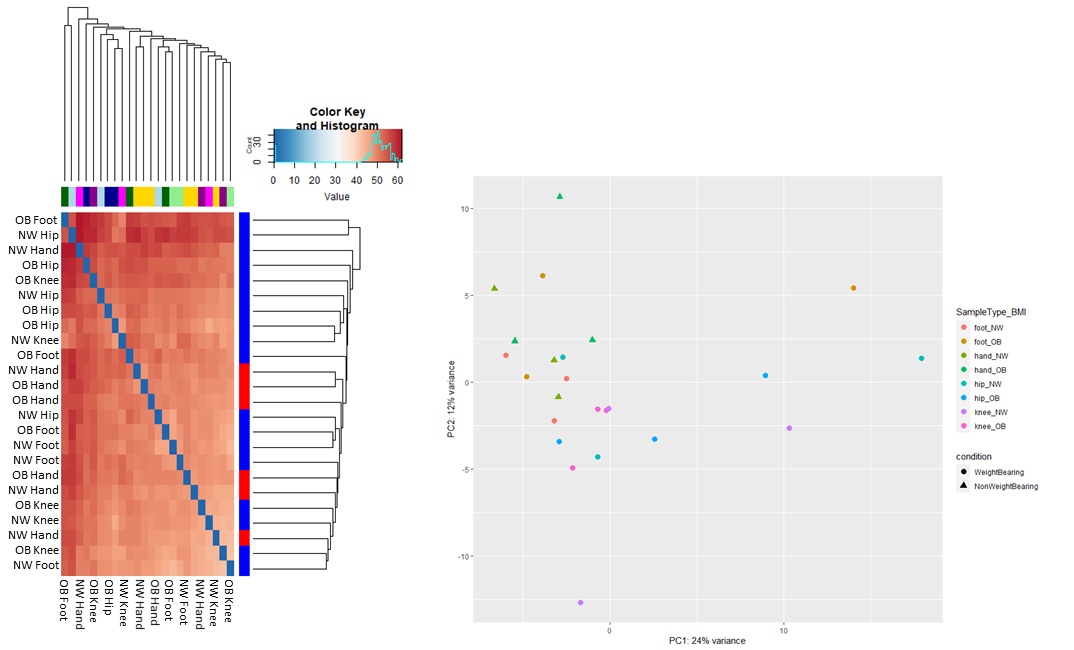


SM.3 – RT-qPCR validations of bulk RNA sequencing data


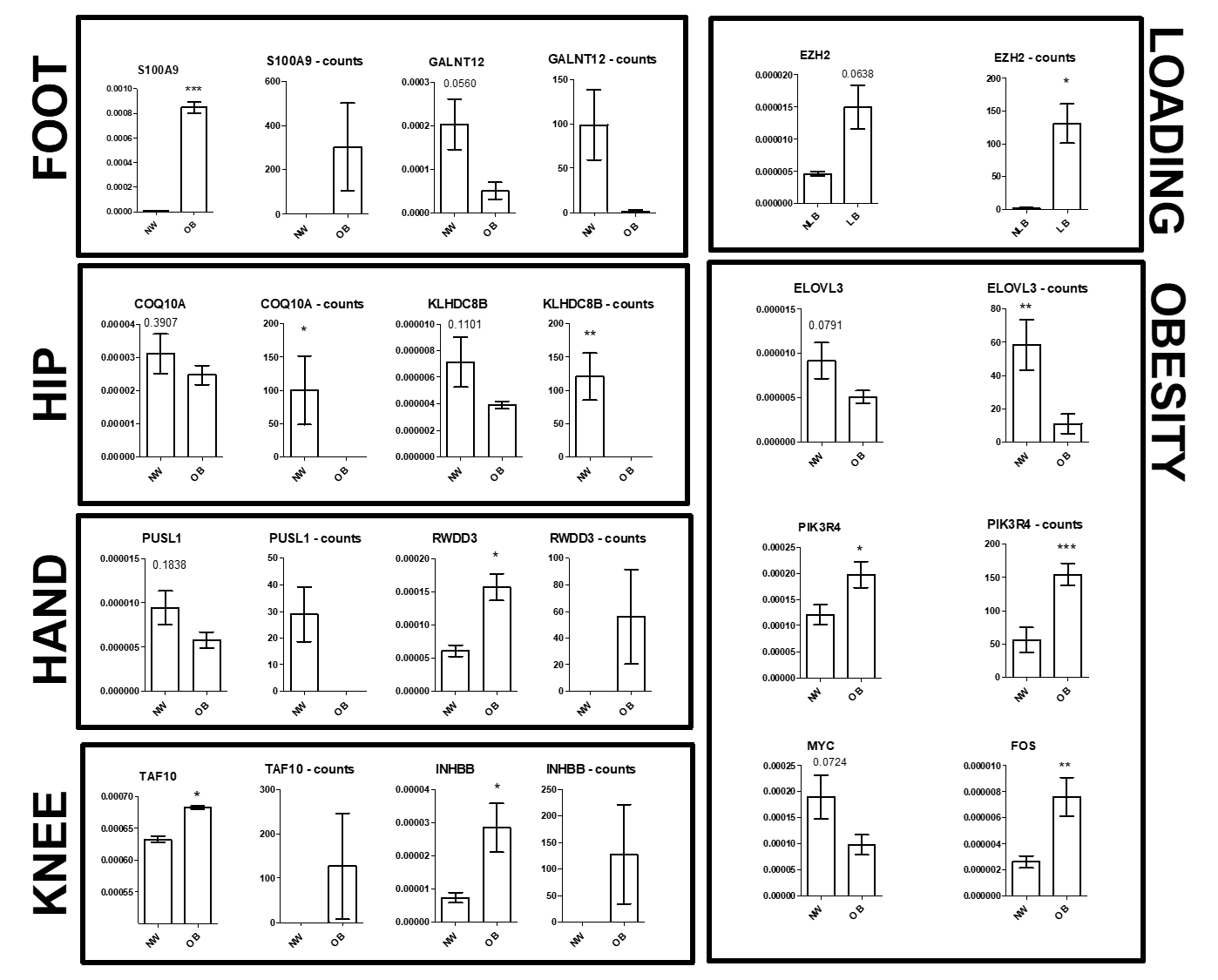


Relative expression is plotted for genes of interest identified in bulk RNA-sequencing of normal-weight and obese OA synovial fibroblasts from loading (hip, knee and foot) and non-loading (hand) joints, along side count data from sequencing. .

Plotted are mean values ± SD and analysed by Student’s t-test. ***P < 0.001, **P < 0.01 and *P < 0.05.

SM.4 - 10X Genomics data Metrics

| Sample | Estimated No. of cells | Mean Reads per Cell | Median Genes per Cell | Total genes Detected | Median UMI Counts per cell | Total number of reads |
| --- | --- | --- | --- | --- | --- | --- |
| Normal Weight | 1092 | 63,679 | 3,128 | 18,901 | 12,241 | 69,538,443 |
| Obese | 1393 | 53,385 | 3,276 | 19,175 | 14,840 | 74,365,820 |

# SM.5 - 10X Genomics data Sample Distribution

Summary of number of cells represented in clusters from each group

| **Cluster** | **Normal Weight** | **Obese** |
| --- | --- | --- |
| **0** | 7 | 503 |
| **1** | 369 | 0 |
| **2** | 343 | 14 |
| **3** | 305 | 2 |
| **4** | 14 | 292 |
| **5** | 2 | 209 |
| **6** | 1 | 194 |
| **7** | 24 | 164 |

# SM.6 - Analysis pipeline and script for 10X Genomics data

#
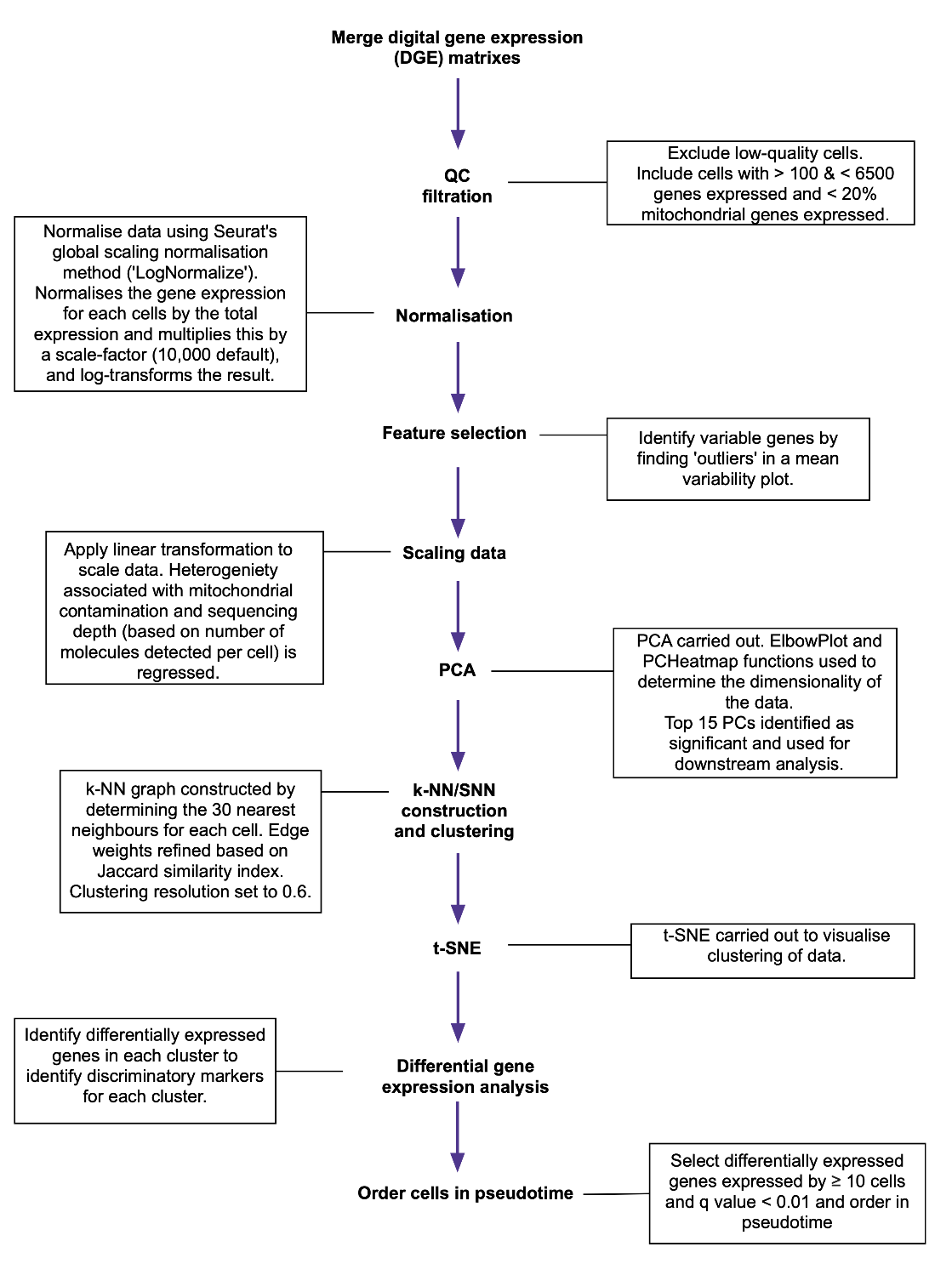


**SM.6 Summary of analysis strategy for single cell RNA sequencing of OA hip-isolated fibroblast samples**. A flow-chart outlining the analysis strategy adopted for single-cell sequencing with scripts below.

# # Load in relevant libraries library(Seurat) library(ggplot2) library(dplyr) library(cowplot) ############ Upload data ############# # Load individual 10X dataset matrix files. # Sample 5- Fibroblasts isolated from the hips of late-disease OA patients (normal-weight patients) sample5.data <- Read10X(data.dir = "~/Desktop/10X_data_matrices/Sample5/filtered_feature_bc_matrix/") sample5 <- CreateSeuratObject(raw.data = sample5.data, project = "Sample5") # Sample 1- Fibroblasts isolated from the hips of late-disease OA patients (obese patients) sample6.data <- Read10X(data.dir = "~/Desktop/10X_data_matrices/Sample6/filtered_feature_bc_matrix/") sample6 <- CreateSeuratObject(raw.data = sample6.data, project = "Sample6") # Merge the two datasets. This is done by appending the barcodes of each matrix file with the particular sample ID. s5s6 <- MergeSeurat(object1 = sample5, object2 = sample6, add.cell.id1 = "s5", add.cell.id2 = "s6", project = "s5s6") # Check that the merged datasets include both of the appended datasets. head(x = s5s6@cell.names) tail(x = s5s6@cell.names) ############ Quality control ############# # Add percentage mitochondrial genes to the metadata of each object ("percent.mito"), by finding genes that start with "MT-" mito.genes <- grep(pattern = "^MT-", x = rownames(x = s5s6@raw.data), value = TRUE) # Calculate the percentage of mitochondrial genes for each barcode. colSum forms column metrics, in this case calculating the % of # mitochondrial genes, by dividing the mito.genes by the overall raw data, for the specific barcode being assessed. percent.mito <- Matrix::colSums(s5s6@raw.data[mito.genes, ]) / Matrix::colSums(s5s6@raw.data) # Add the percent.mito to the metadata and call the column "percent.mito". s5s6 <- AddMetaData(object = s5s6, metadata = percent.mito, col.name = "percent.mito") # Visualize QC metrics as a violin plot VlnPlot(object = s5s6, features.plot = c("nGene", "nUMI", "percent.mito"), nCol = 3) # Filtering the data based on determined quality control parameters s5s6 <- FilterCells(object = s5s6, subset.names = c("nGene", "percent.mito"), low.thresholds = c(100, -Inf), high.thresholds = c(6200, 0.2)) # Check how many cells remain post filtration. s5s6 ############ Normalisation ############# s5s6 <- NormalizeData(object = s5s6, normalization.method = "LogNormalize", scale.factor = 10000) ############ Feature selection ############# # Find variable genes s5s6 <- FindVariableGenes(object = s5s6, mean.function = ExpMean, dispersion.function = LogVMR, x.low.cutoff = 0.0125, x.high.cutoff = 5, y.cutoff = 2) # Print number of variable genes identified length(s5s6@var.genes) ############ Scale data ############# # Scaling data and removing unwanted sources of variation s5s6 <- ScaleData(object = s5s6, vars.to.regress = c("nUMI", "percent.mito")) # Check number of cells passed for PCA analysis s5s6 ############ Assess dimensionality of the data ############# # Perform PCA on the scaled data s5s6 <- RunPCA(object = s5s6, pc.genes = s5s6@var.genes, do.print = TRUE, pcs.print = 1:5, genes.print = 5) # Visualise PCA PCAPlot(object = s5s6, dim.1 = 1, dim.2 = 2) # Run PCElbowPlot function to assess dimentionality # This function ranks the principal components based on the percentage of variance explained by each component PCElbowPlot(object = s5s6) ############ Cluster cells ############# # Before clustering the cells, run the 'RunTSNE' and 'TSNEPlot' functions to be able to see how the cells cluster according to sample s5s6 <- RunTSNE(object = s5s6, dims.use = 1:15, do.fast = TRUE) TSNEPlot(object = s5s6, do.label = F, pt.size = 2, colors.use = c("royalblue2", "indianred1")) # Cluster the cells. # Construct kNN/SNN graphs based on dimensionality identified by PCA (top 15 PCs). # Cluster cells based on Louvain algorithm with a resolution of 0.6 s5s6 <- FindClusters(object = s5s6, reduction.type = "pca", dims.use = 1:15, resolution = 0.6, print.output = 0, save.SNN = TRUE, force.recalc = T) # Run t-SNE dimensional reduction based on top 15 PCs s5s6 <- RunTSNE(object = s5s6, dims.use = 1:15, do.fast = TRUE) # Visualise clustering results (t-SNE visualisation) TSNEPlot(object = s5s6, do.label = T, pt.size = 2) ########### Differential gene expression analysis ############### # Find all markers for each cluster using Wilcoxon Rank Sum test. Return all DEGs in each cluster (genes expressed # in at least 25% of cells), compared to the rest. Export results into a csv file. # Cluster 0 cluster0.markers <- FindMarkers(object = s5s6, ident.1 = 0, min.pct = 0.25) write.table(cluster0.markers, file = "s5s6_Cluster0_markers.csv", sep = ",", row.names = TRUE, col.names = NA) # Cluster 1 cluster1.markers <- FindMarkers(object = s5s6, ident.1 = 1, min.pct = 0.25) write.table(cluster1.markers, file = "s5s6_Cluster1_markers.csv", sep = ",", row.names = TRUE, col.names = NA) # Cluster 2 cluster2.markers <- FindMarkers(object = s5s6, ident.1 = 2, min.pct = 0.25) write.table(cluster2.markers, file = "s5s6_Cluster2_markers.csv", sep = ",", row.names = TRUE, col.names = NA) # Cluster 3 cluster3.markers <- FindMarkers(object = s5s6, ident.1 = 3, min.pct = 0.25) write.table(cluster3.markers, file = "s5s6_Cluster3_markers.csv", sep = ",", row.names = TRUE, col.names = NA) # find all markers of cluster 4 cluster4.markers <- FindMarkers(object = s5s6, ident.1 = 4, min.pct = 0.25) write.table(cluster4.markers, file = "s5s6_Cluster4_markers.csv", sep = ",", row.names = TRUE, col.names = NA) # find all markers of cluster 5 cluster5.markers <- FindMarkers(object = s5s6, ident.1 = 5, min.pct = 0.25) print(x = head(x = cluster5.markers, n = 5)) write.table(cluster5.markers, file = "s5s6_s5s6_Cluster5 markers.csv", sep = ",", row.names = TRUE, col.names = NA) # find all markers of cluster 6 cluster6.markers <- FindMarkers(object = s5s6, ident.1 = 6, min.pct = 0.25) write.table(cluster6.markers, file = "s5s6_Cluster6_markers.csv", sep = ",", row.names = TRUE, col.names = NA) # find all markers of cluster 7 cluster7.markers <- FindMarkers(object = s5s6, ident.1 = 7, min.pct = 0.25) write.table(cluster7.markers, file = "s5s6_Cluster7_markers.csv", sep = ",", row.names = TRUE, col.names = NA) # Find markers for every cluster compared to all remaining cells, report only the positive ones. Export results as csv files. s5s6_pos.markers <- FindAllMarkers(object = s5s6, only.pos = TRUE, min.pct = 0.25, thresh.use = 0.25) write.table(s5s6_pos.markers, file = "Markers_for_every_cluster_onlypos.csv", sep = ",", row.names = TRUE, col.names = NA) #### Create a heatmap displaying the top 10 genes for each cluster based on average log fold change # Identify the top 10 most differentially expressed genes based on average log fold change; order according to average log fold change. top10 <- s5s6_pos.markers %>% group_by(cluster) %>% top_n(10, avg_logFC) # Construct heatmap.Setting slim.col.label to TRUE will print just the cluster IDS instead of # every cell name DoHeatmap(object = s5s6, genes.use = top10$gene, slim.col.label = TRUE, remove.key = F) ######### Assess DEGs between each sample ID # Change the object's identity class to "orig.ident" instead of clusters s5s6 <- SetAllIdent(object = s5s6, id = "orig.ident") # Find differentially expressed genes between the different sample IDs and export results as a csv file. markers <- FindAllMarkers(object = s5s6) write.table(markers, file = "s5s6_DEGs.csv", sep = ",", row.names = TRUE, col.names = NA) ########### Data visualisation ############### # VlnPlots to visualise expression of genes of interest- grouped by sample ID VlnPlot(object = s5s6, features.plot = c("GPX3", "CXCL14"), group.by = "orig.ident") # Feature plots to visualise expression of genes of interest FeaturePlot(object = s5s6, features.plot = c("SLC39A8", "PLTP"), cols.use = c("grey", "blue"), reduction.use = "tsne") ######################################## MONOCLE ANALYSIS ###################################################### ###### Trajectory analysis ###### # Load monocle library library(monocle) # Import object from Seurat into monocle as a CDS (cell dataset) importCDS(s5s6, import_all = T) # Check that the cell names have been annotatded with the particular identifier head(x = s5s6@cell.names) tail(x = s5s6@cell.names) # Check the cell numbers for each sample. table(s5s6@meta.data$orig.ident) # Convert to imported data to monocle CDS s5s6.monocle <- importCDS(s5s6, import_all = TRUE) # Check the phenotypic data. Ensure that cell names have been annotated with the unique identifier. Also, ensure that there is a column # for cell identity (i.e. a column called orig.ident) head(pData(s5s6.monocle)) tail(pData(s5s6.monocle)) # Examine expression data. Number of genes across number of single cells. Double check that import is correct. dim(exprs(s5s6.monocle)) # Check the feature information - number of genes. Double check that import is correct. dim(fData(s5s6.monocle)) # Ensure that there is a column called gene_short_name head(fData(s5s6.monocle)) # Load dataset into monocle's main class "newCellDataSet" my_cds <- newCellDataSet(exprs(s5s6.monocle), phenoData = new("AnnotatedDataFrame", data = pData(s5s6.monocle)), # phenotype data featureData = new("AnnotatedDataFrame", data = fData(s5s6.monocle)), # feature data lowerDetectionLimit = 0.5, expressionFamily = negbinomial.size()) # Check the cell dataset- information on dataset my_cds # Perform normalisation and variance estimation steps. my_cds <- estimateSizeFactors(my_cds) my_cds <- estimateDispersions(my_cds) # Run the detectGenes() function, to tally the number of cells expressing a gene and the number of genes expressed among all cells my_cds <- detectGenes(my_cds) # The detectGenes function adds column 'num_cells_expressed'- this column tallies the number of cells # expressing a particular gene (a gene is “expressed” if there is at least one count). head(fData(my_cds)) tail(fData(my_cds)) # A summary of the num_cells_expressed column summary(fData(my_cds)$num_cells_expressed) # The number of genes expressed (num_genes_expressed) per cell is stored in phenoData. # Note that if a gene has 10 UMIs or 1 UMI, it is still tallied as 1. head(pData(my_cds)) tail(pData(my_cds)) # Standardise to Z-distribution- i.e. normal distribution where mean is equal to zero and SD is equal to one. x <- pData(my_cds)$num_genes_expressed x_1 <- (x - mean(x)) / sd(x) summary(x_1) # Assess distribution of gene expression. y-axis is expression count, x-axis is standard deviation. # Monocle calculates mean total expression/cell and the standard deviation. Then takes the mean +/- 2 * SD. df <- data.frame(x = x_1) ggplot(df, aes(x)) + geom_histogram(bins = 50) + geom_vline(xintercept = c(-2, 2), linetype = "dotted", color = 'red') # Looking at UMI count vs. num_genes_expressed. This allows us to potentially be able to identify any doublets as these will have a # higher gene expression and UMI count (nUMI). ggplot(pData(my_cds), aes(num_genes_expressed, nUMI)) + geom_point() # Next we select the most highly variable genes, based on average expression and variability acrss the cells. # The dispersionTable() function calculates the mean and dispersion values. disp_table <- dispersionTable(my_cds) head(disp_table) # Select genes for ordering- select particular genes that are expressed by at least 10 cells. print(head(fData(my_cds))) expressed_genes <- row.names(subset(fData(my_cds), num_cells_expressed >= 10)) # "differentialGeneTest" tests each gene for differential expression as a function of pseudotime or # according to other covariates as specified (in this case, orig.ident) diff_test_res <- differentialGeneTest(my_cds[expressed_genes,], fullModelFormulaStr = "~orig.ident") # Based on the genes identified from the "differentialGeneTest", we select the genes with a qvalue of less than 0.01 # These get stored as a set of genes in a list called "ordering_genes". ordering_genes <- row.names (subset(diff_test_res, qval < 0.01)) table (ordering_genes) # The 'setOrderingFilter function marks genes that will be used for downstream in pseudotime analysis my_cds <- setOrderingFilter(my_cds, ordering_genes) # The plot_ordering_genes function shows how variability (dispersion) in a gene's expression depends on the average expression across cells. # The red line shows Monocle's expectation of the dispersion based on this relationship. # The genes we marked for use in downstream analysis are shown as black dots, while the others are shown # as grey dots. plot_ordering_genes(my_cds) # Next, the space is reduced down to two dimensions (using the 'DDRTree' method) my_cds <- reduceDimension(my_cds, max_components = 2, method = 'DDRTree') # Now that the space has been reduced, we use the "orderCells" function which learns a "trajectory" and calculates where each cell falls within it. # This function takes as input a CellDataSet and returns it with two new columns: # Pseudotime and State, which together encode where each cell maps to the trajectory my_cds <- orderCells(my_cds) # Check the phenotypic data for the object to ensure that a column for Pseudotime and State have been added. head(pData(my_cds)) # Plot trajectory based on cell clustering plot_cell_trajectory(my_cds, color_by = "res.0.6") # Plot trajectory based on "orig.ident" (i.e sampleID) plot_cell_trajectory(my_cds, color_by = "orig.ident") # Plot trajectory based on Pseudotime. plot_cell_trajectory(my_cds, color_by = "Pseudotime") # Assess expression of particular genes ("blast_genes") across the different states blast_genes <- row.names(subset(fData(my_cds), gene_short_name %in% c("CHI3L1", "VCAM1", "IGFBP7"))) # Check how gene expression changes with pseudotime- according to sampleID and clusters plot_genes_in_pseudotime(my_cds[blast_genes], color_by = "orig.ident") plot_genes_in_pseudotime(my_cds[blast_genes], color_by = "res.0.6")

Fig. S1.


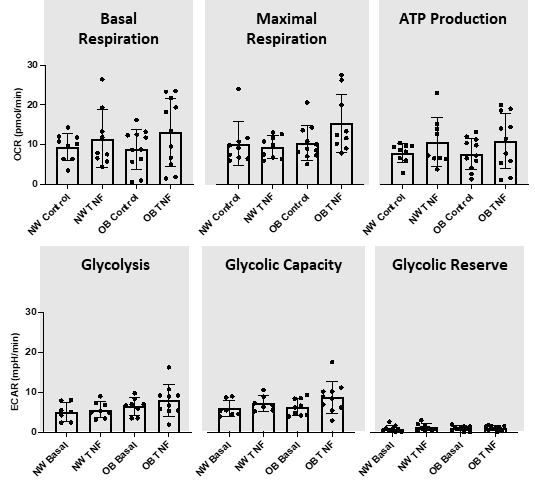


**Fig. S1. Graphical summary of mitochondrial respiration and glycolysis of obese and normal weight OA joint synovial fibroblasts.** Seahorse analysis was used to measure oxygen consumption rate (OCR, top panel) and extracellular acidification rate (ECAR, bottom panel) of obese (n=) and normal weight (n=) synovial fibroblasts to determine mitochondrial respiration and glycolysis, respectively. Two-way ANOVA with Tukey HSD post-hoc test for different joints and stimulation. Plotted are mean values ± SD.

Fig. S2.


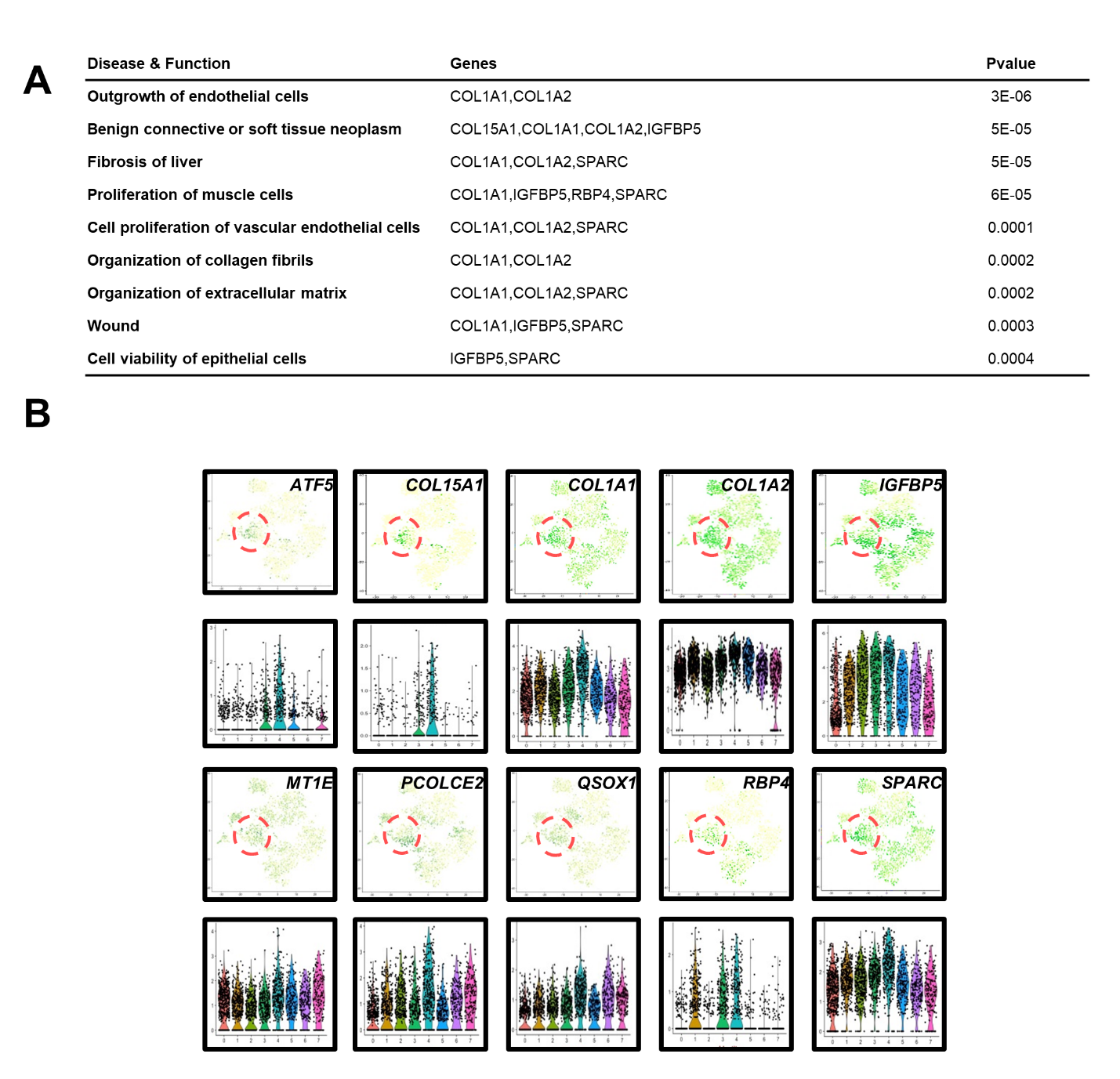


Fig. S2. Summary of DEGs in Cluster 4. A) List of Cluster 4 genes enriched to each functional pathway from IPA analysis highlighting genes with define the clusters. B) Feature plots displaying expression of Cluster 4 specific markers on the t-SNE map along with violin plots showing the expression levels of these markers for each cluster. Genes displayed are representative of genes presented in heatmap Figure 3C.

Fig. S3.


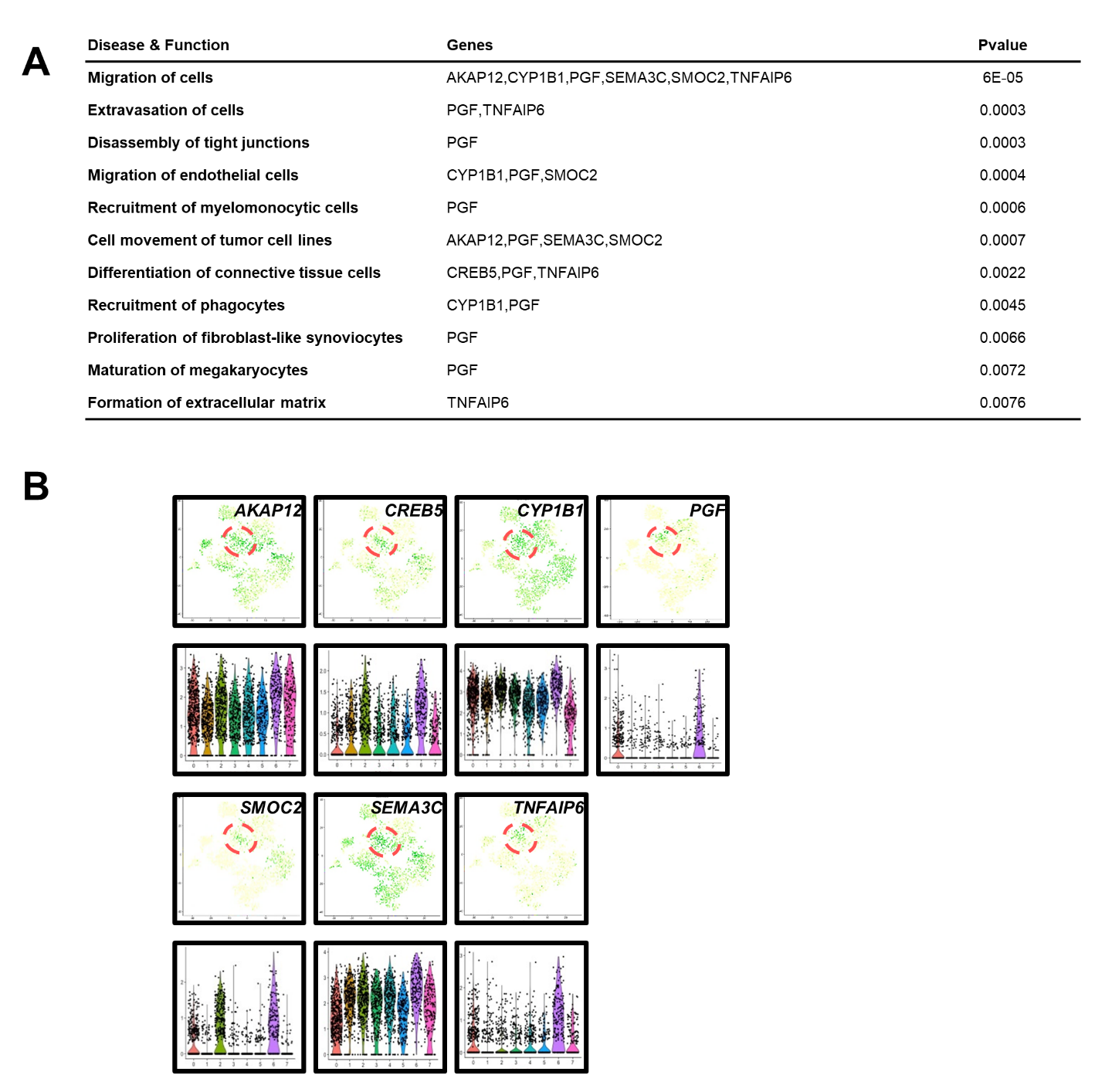


Fig. S3. Summary of DEGs in Cluster 6. A) List of Cluster 6 genes enriched to each functional pathway from IPA analysis highlighting genes with define the clusters. B) Feature plots displaying expression of Cluster 6 specific markers on the t-SNE map along with violin plots showing the expression levels of these markers for each cluster. Genes displayed are representative of genes presented in heatmap Figure 3C.

Fig. S4.


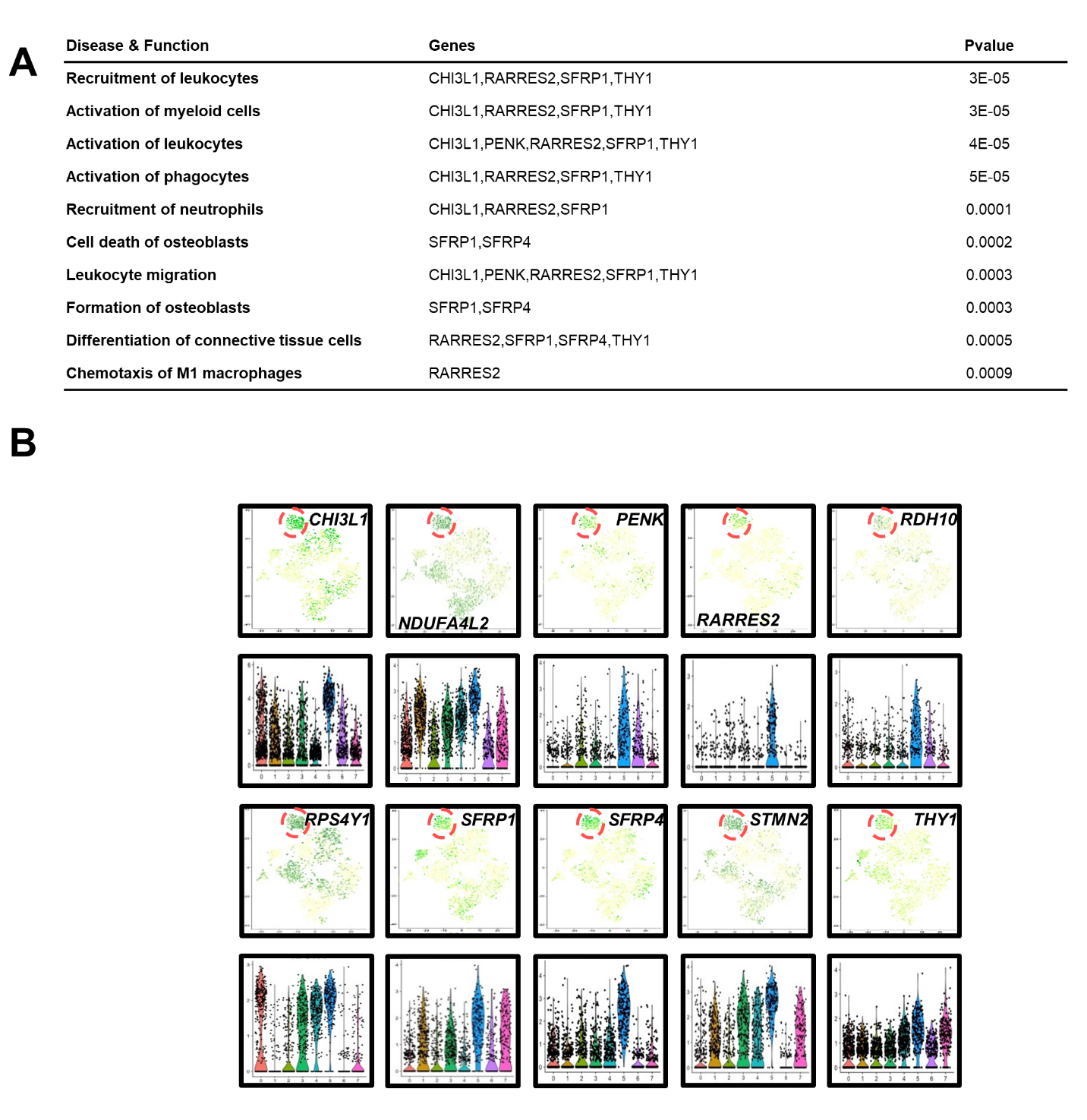


Fig. S4. Summary of DEGs in Cluster 5. A) List of Cluster 5 genes enriched to each functional pathway from IPA analysis highlighting genes with define the clusters. B) Feature plots displaying expression of Cluster 5 specific markers on the t-SNE map along with violin plots showing the expression levels of these markers for each cluster. Genes displayed are representative of genes presented in heatmap Figure 3C.

Fig. S5.


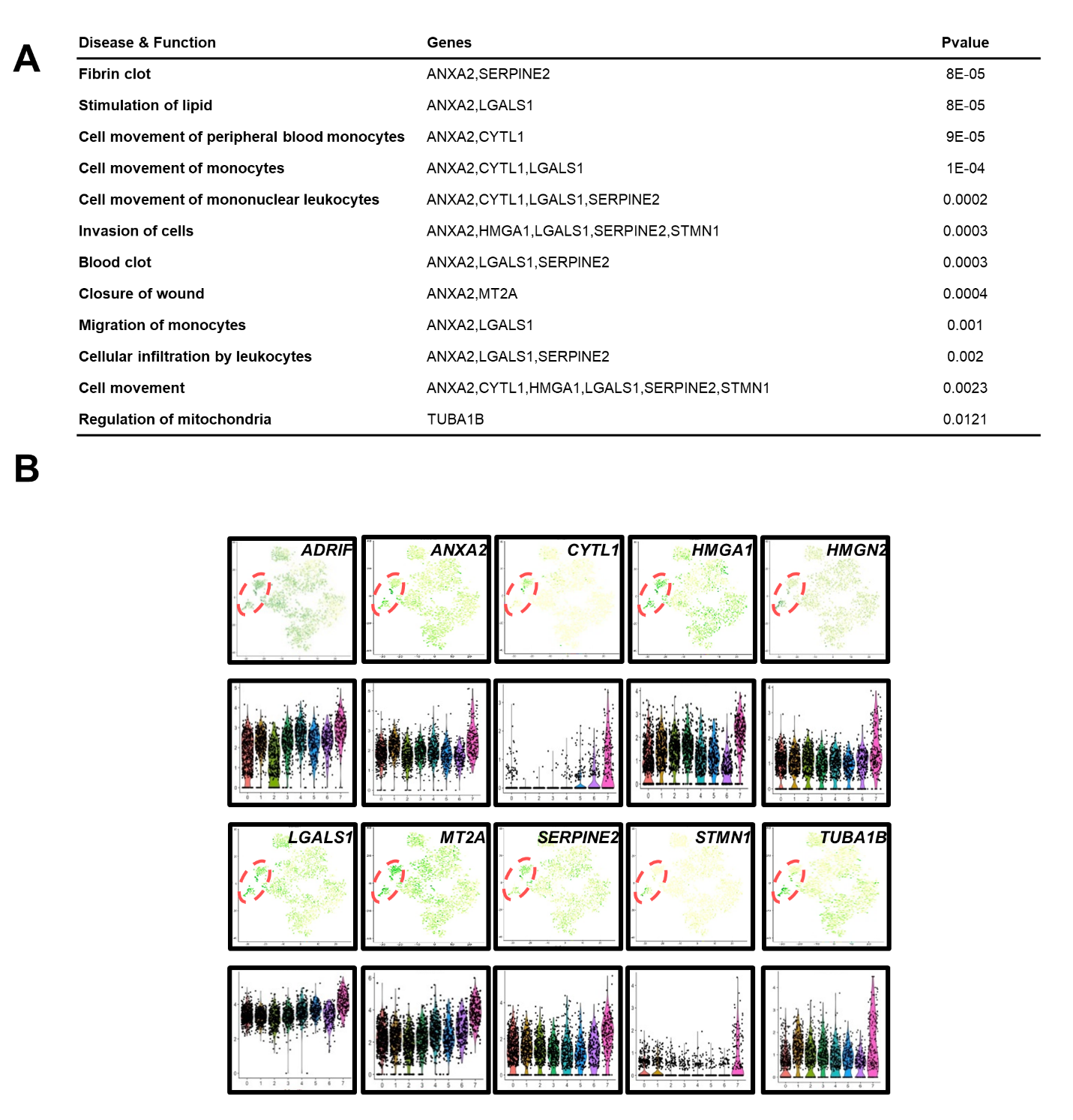


Fig. S5. Summary of DEGs in Cluster 7. A) List of Cluster 7 genes enriched to each functional pathway from IPA analysis highlighting genes with define the clusters. B) Feature plots displaying expression of Cluster 7 specific markers on the t-SNE map along with violin plots showing the expression levels of these markers for each cluster. Genes displayed are representative of genes presented in heatmap Figure 3C.

Fig. S6.


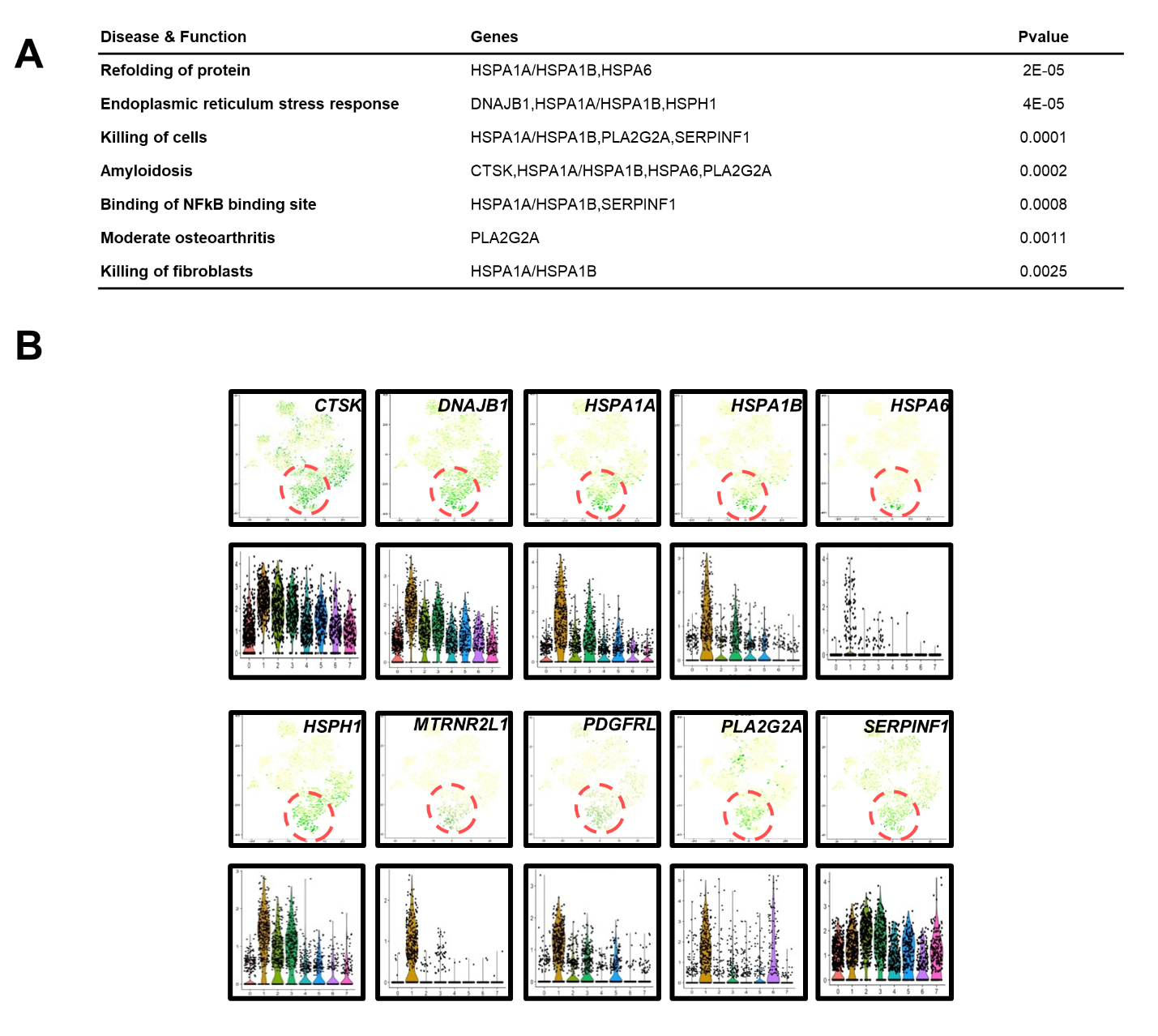


Fig. S6. Summary of DEGs in Cluster 1. A) List of Cluster 1 genes enriched to each functional pathway from IPA analysis highlighting genes with define the clusters. B) Feature plots displaying expression of Cluster 1 specific markers on the t-SNE map along with violin plots showing the expression levels of these markers for each cluster. Genes displayed are representative of genes presented in heatmap Figure 3C.

Fig. S7.


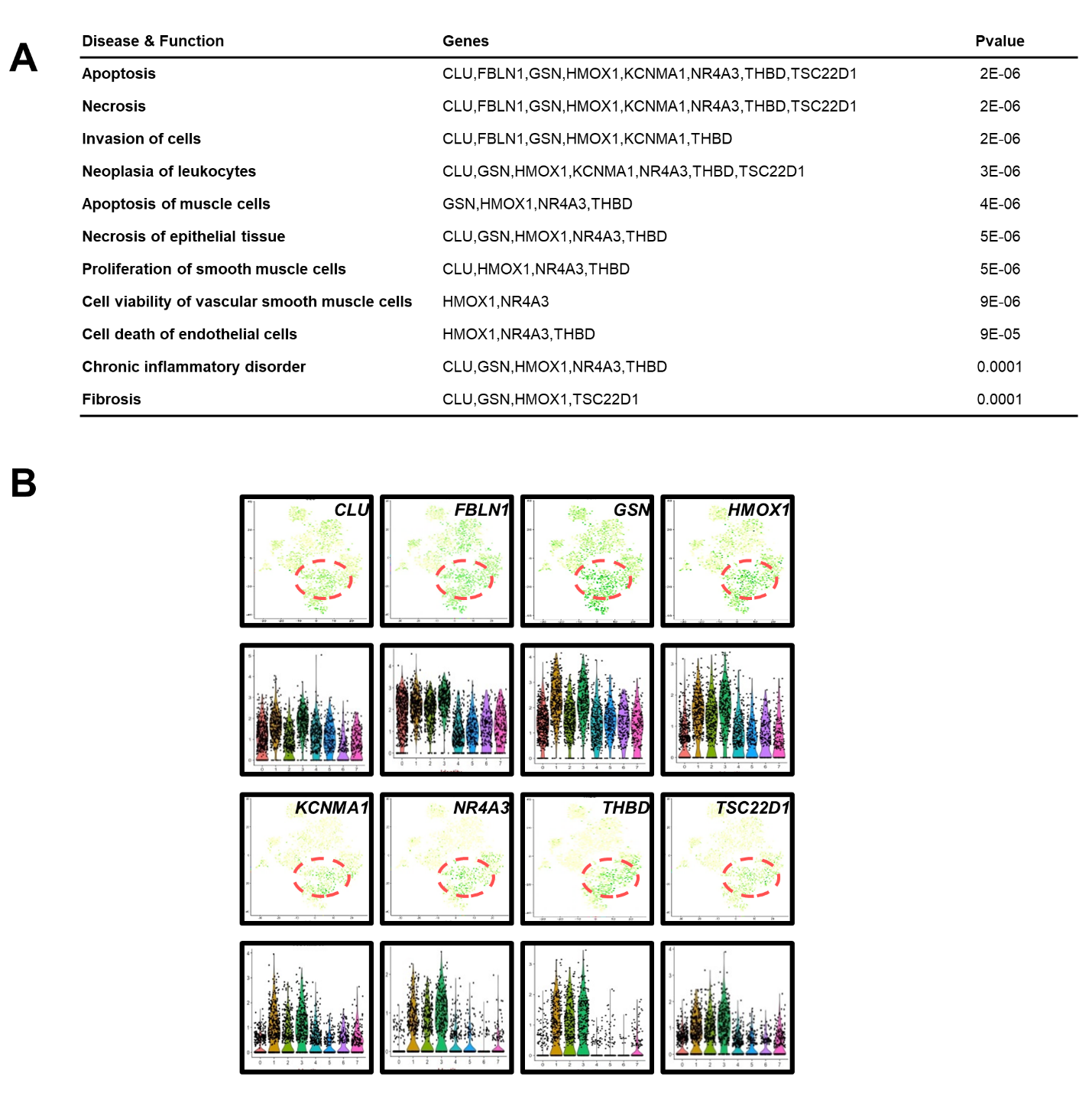


Fig. S7. Summary of DEGs in Cluster 3. A) List of Cluster 3 genes enriched to each functional pathway from IPA analysis highlighting genes with define the clusters. B) Feature plots displaying expression of Cluster 3 specific markers on the t-SNE map along with violin plots showing the expression levels of these markers for each cluster. Genes displayed are representative of genes presented in heatmap Figure 3C.

Fig. S8.


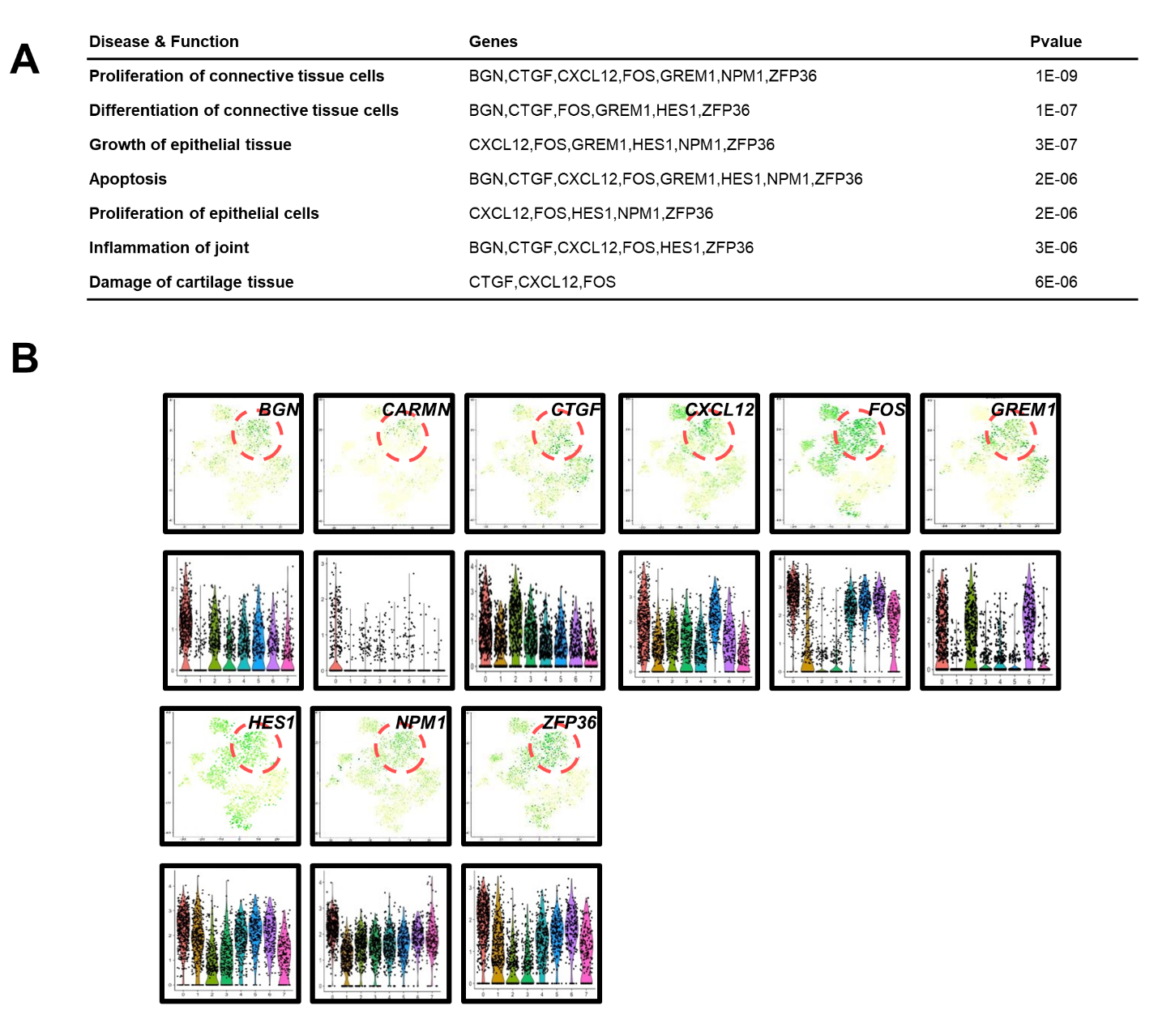


Fig. S8. Summary of DEGs in Cluster 0. A) List of Cluster 0 genes enriched to each functional pathway from IPA analysis highlighting genes with define the clusters. B) Feature plots displaying expression of Cluster 0 specific markers on the t-SNE map along with violin plots showing the expression levels of these markers for each cluster. Genes displayed are representative of genes presented in heatmap Figure 3C.

Fig. S9.


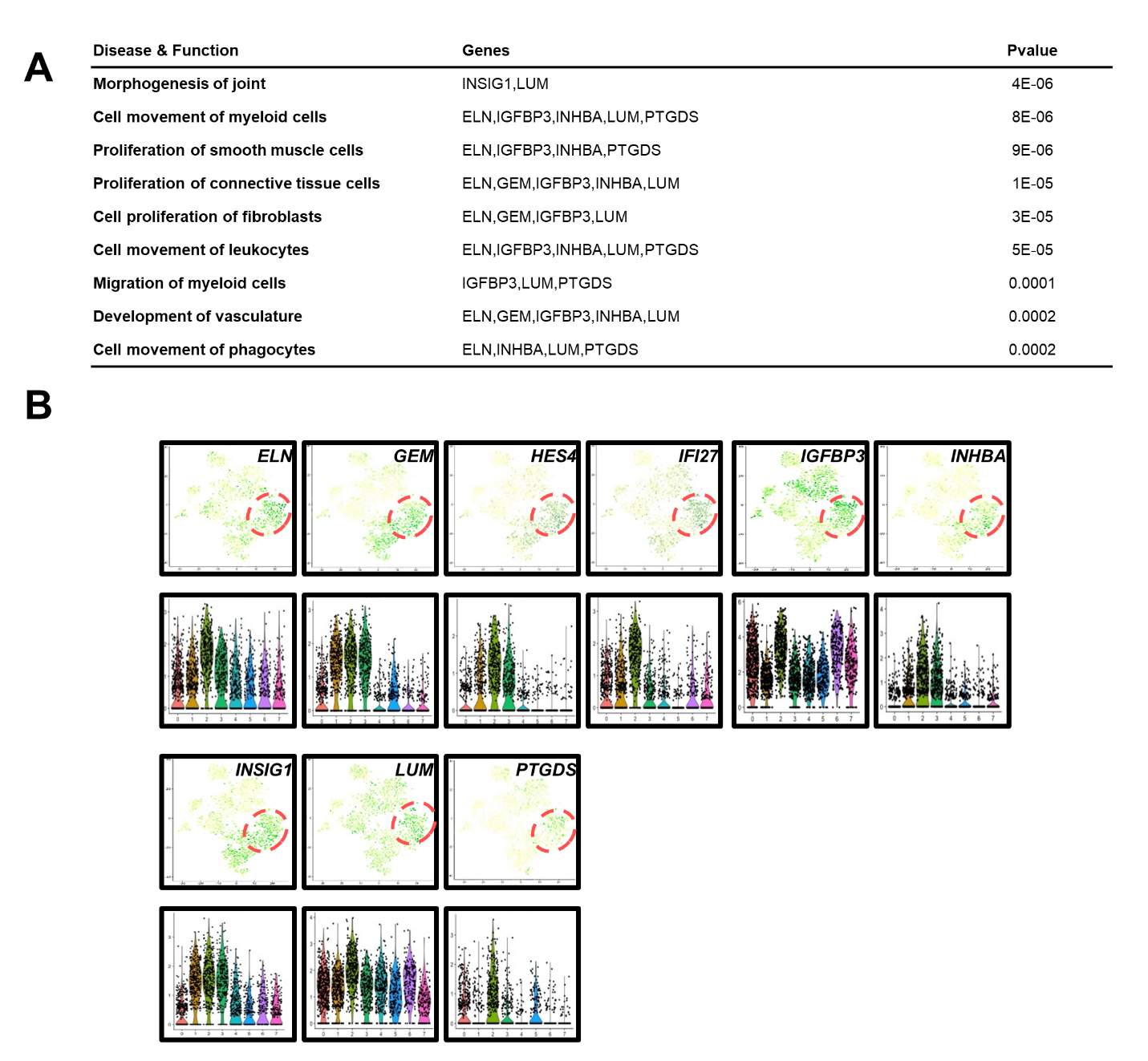


Fig. S9. Summary of DEGs in Cluster 2. A) List of Cluster 2 genes enriched to each functional pathway from IPA analysis highlighting genes with define the clusters. B) Feature plots displaying expression of Cluster 2 specific markers on the t-SNE map along with violin plots showing the expression levels of these markers for each cluster. Genes displayed are representative of genes presented in heatmap Figure 3C.

Fig. S10.


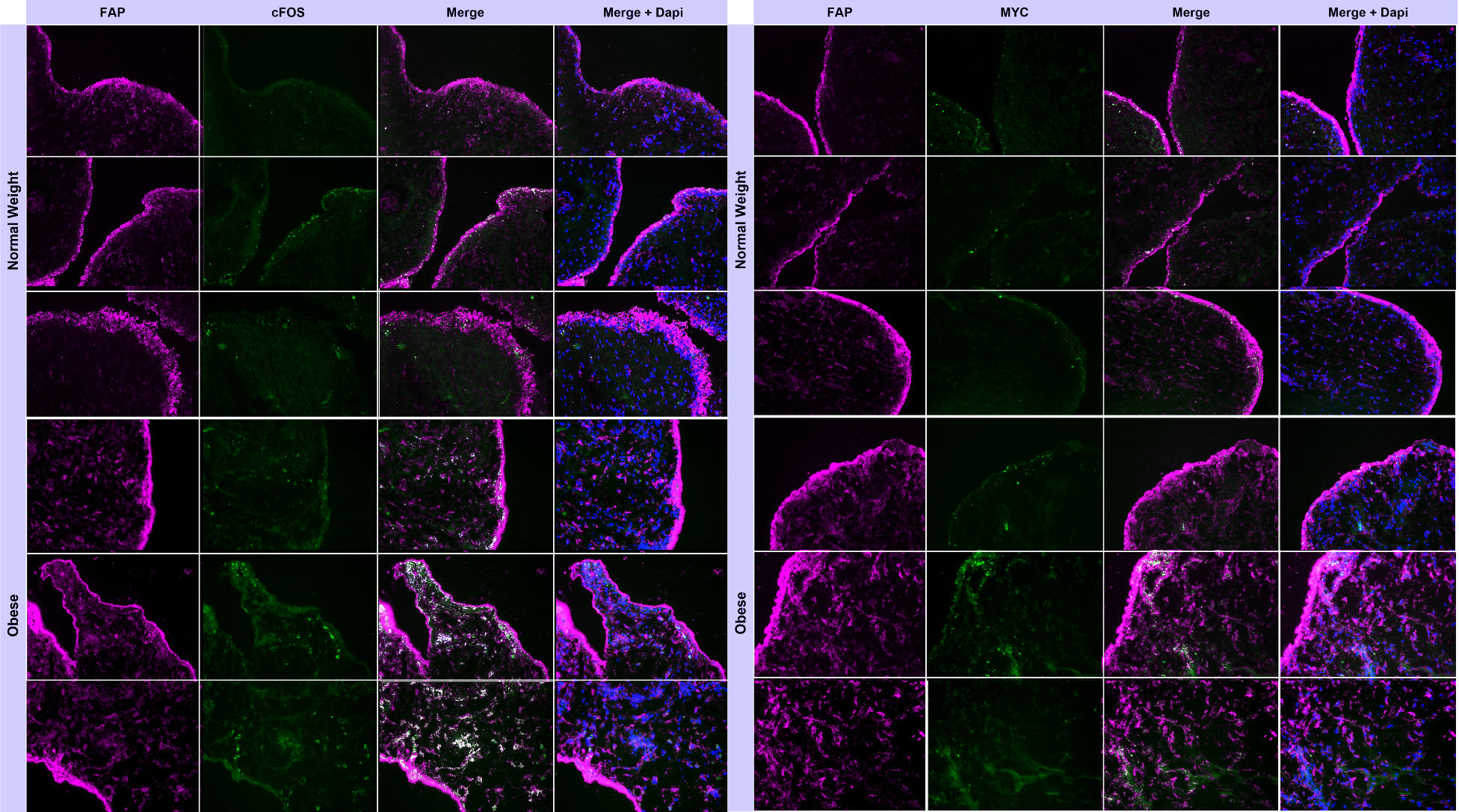


Fig. S10. Pseudo-coloured accessible IF panel. Representative immunofluorescence imaging for c-Fos (green, left panel) and c-Myc (green, right panel) in OA hip synovial tissue from normal weight (NW) and obese (OB) patients. SF were visualized with FAP (pseudo magenta), and nuclei were stained with DAPI (blue), colocalization has been pseudo-coloured white.
